# Minimalist Social-Affective Value for Use in Joint Action: A Neural-Computational Hypothesis

**DOI:** 10.3389/fncom.2016.00088

**Published:** 2016-08-23

**Authors:** Robert Lowe, Alexander Almér, Gustaf Lindblad, Pierre Gander, John Michael, Cordula Vesper

**Affiliations:** ^1^Ice Lab, Applied IT, University of GothenburgGothenburg, Sweden; ^2^Interaction Lab, School of Informatics, University of SkövdeSkövde, Sweden; ^3^Department of Cognitive Science, Central European UniversityBudapest, Hungary

**Keywords:** emotions, associative two-process theory, social value computation, joint action, minimal architectures, social Aff-ATP hypothesis, extended common currency

## Abstract

Joint Action is typically described as social interaction that requires coordination among two or more co-actors in order to achieve a common goal. In this article, we put forward a hypothesis for the existence of a neural-computational mechanism of affective valuation that may be critically exploited in Joint Action. Such a mechanism would serve to facilitate coordination between co-actors permitting a reduction of required information. Our hypothesized affective mechanism provides a value function based implementation of Associative Two-Process (ATP) theory that entails the classification of external stimuli according to outcome expectancies. This approach has been used to describe animal and human action that concerns differential outcome expectancies. Until now it has not been applied to social interaction. We describe our Affective ATP model as applied to social learning consistent with an “extended common currency” perspective in the social neuroscience literature. We contrast this to an alternative mechanism that provides an example implementation of the so-called social-specific value perspective. In brief, our Social-Affective ATP mechanism builds upon established formalisms for reinforcement learning (temporal difference learning models) nuanced to accommodate expectations (consistent with ATP theory) and extended to integrate non-social and social cues for use in Joint Action.

## Introduction

The notion of *Joint Action* has received various definitions. The popular perspective from the 90s onwards has viewed it as a manifestation of shared intentions to act between two or more individuals (e.g., Gilbert, 1990; Searle, 1990; Bratman, 1992; Tuomela, 1993). Tomasello, for example, has stated: “[t]he sine qua non of collaborative action is a joint goal and a joint commitment” (Tomasello, 2010, p. 181). Bratman's (1992) shared intentional position on Joint Action can be described accordingly: (i) inter-acting agents have intentional behavior toward an outcome, (ii) agents adjust (“mesh”) subplans of the intentional behavior to account for the other(s), and (iii) the agents are aware of the (adjusting) intentions of the other(s). The collective aspect is supposedly captured by this form, and there is a requirement of interrelatedness of individual intentions among group members: group members have the “same” individual thought on this collective form. An important ingredient in Tuomela's social ontology, for example, is the collective mode of thought (we-mode) to be distinguished from the me-mode. In this view, broadly, individuals can act as members of groups either for satisfying private ends or for satisfying group ends.

Definitions abound that attempt to de-emphasize the role that shared intentions play in Joint Action, many of which are action- or outcome-focused. Butterfill (2012), for example, bases his definition of Joint Action on shared goals rather than shared intentions. On this account, goal-directed^1^ behavior need not be intentional “there are ways of representing actions as goal-directed which do not involve representing intentions or any other propositional attitudes of agents” (p. 13). Other “minimalist” accounts of Joint Action can be found. For example, Miller (1992) posits that in Joint Action: “there is more than one agent; each agent is performing (at least) one action; each agent's action is dependent on the actions of the other agents” (p. 275). Knoblich and Jordan (2002) define Joint Action as a: “[situation] where neither member of a group can achieve a common goal on his own but only with the help of the other member” (p. 2) and Sebanz et al. (2006), describe Joint Action as: “any form of social interaction whereby two or more individuals coordinate their actions in space and time to bring about a change in the environment” (p. 70).

These more “minimalist,” action/goal-oriented perspectives focus on those mechanisms that are requisite to many Joint Actions of the type that require coordination in time and space. Minimal mechanisms are pertinent when tasks are new to the actors and/or challenging (not mastered): “minimal representations may be formed when a novice learns to perform a new joint action, particularly if the novice's task is cognitively demanding and leaves insufficient resources to form rich representations that include all components of the joint action” (Loehr and Vesper, 2016, p. 536). It is an open question as to what extent such non-mental mechanisms underlie, constrain, or even substitute for the “higher” cognitive mentalizing purported by the advocates of shared intentionality in Joint Action.

The remainder of this article breaks down as follows: In Section Minimal Mechanisms and Coordination “Smoothers” in Joint Action we discuss minimalist mechanisms that enable Joint Action. Section An Affective Account of Associative Two-Process Theory concerns a description of a value function based on ATP theory, which has been applied to individual learning of differential affective states. In this section, we also introduce our (novel) hypothesis suggesting that such an “affective” implementation of ATP may be applied to a social context relevant to Joint Action. We call this the *Social Aff-ATP hypothesis*. In Section Neural-Computational Basis for Affective Valuation, we describe our existing neural-computational account of ATP as it applies to the *individual*, and then propose the (neural-computational) mechanisms that underlie our *Social Aff-ATP hypothesis*. Finally, in Section Discussion we provide a discussion of the mechanism's functional relevance to a Joint Action context.

## Minimal mechanisms and coordination “Smoothers” in joint action

### Investigating minimal mechanisms of joint action

The notion of minimalism appeals to evolutionary (Tomasello et al., 2005; de Waal, 2008; Decety, 2011) and developmental (Milward et al., 2014; Milward and Sebanz, 2016; Steinbeis, 2016) continuity regarding the mechanisms applicable to social interaction. It provides a bottom-up approach, which attempts to minimize assumptions about the cognitive mechanisms that may account for a particular behavioral phenomenon. *Minimalization* is closely related to fundamental imperatives to minimize the complexity of Joint Action in Bayesian or active inference treatments of communication and neural hermeneutics (Frith and Wentzer, 2013; Friston and Frith, 2015). Here, the idea is to minimize the likelihood of forward models of self and other; where the marginal likelihood (or model evidence) is equal to accuracy minus complexity. This means that optimal exchange and Joint Action should be minimally complex and as “smooth” as possible. Thereby, a minimalist approach can be seen as a perspective that fosters deeper understanding of the origins and functions of processes that underlie, or contribute to, performance of Joint Actions.

A typical “minimalist” Joint Action example is given in the form of a table-moving scenario. Two individuals are said to have as their goal to move a table from place A to B (cf. Sebanz et al., 2006). The table may be too heavy for one actor but manageable for the two actors. This example requires that the actors continually take into account, and adjust to, the patterns of sensorimotor activity of the other. The actors must not simply react to the actions of the other but also predict the other's actions and adapt to them in order to best realize the achieving of the common goal.

A number of studies have sought to investigate the minimal mechanisms that may underlie different varieties of Joint Action (cf. Sebanz et al., 2003, 2005; Richardson et al., 2012). In such settings “representing” task-based states of others (action outcomes, task rules) are not required for successful completion of the joint *activity*^2^. The indication of presence of such representations, however, is suggestive of their ubiquity and general applicability in social interactions. Apparently, individuals can't help but represent the spatiotemporally coincident (or overlapping) activities of others. The work by Sebanz et al. (2003) and Sebanz et al. (2005), has, respectively, inferred the existence of action-based, and task-based, representations of others according to scenarios that entailed joint activity where the successful completion of the task for either individual did not depend on the performance of the other in the task.

Atmaca et al. (2011), similar to the findings of Sebanz and colleagues above, found that subjects will represent task rules of another co-acting participant even when such knowledge does not beneficially impact upon performance. The general finding of Atmaca et al. (2011) was that participants produced a bigger difference in reaction times when responding to incompatible, vs. compatible, stimuli when they were in a joint condition (another participant present) compared to when they were in the individual condition. The experimenters also found that it was important as to whether participants believed that the “other” in the joint condition acted intentionally. As for the Sebanz et al. (2005) experiment, Atmaca et al. (2011) concluded that people in a *Joint Activity* setting have a strong tendency to represent the task (stimulus-response, or S-R, mappings) of others even when it is not required for successful completion of the task.

The above examples provide evidence that humans can't help but represent information about others when it concerns actions and (arbitrary) task rules using simple stimulus-response mappings. Such tendenices may bring to bear on, or have even evolved in the context of, Joint Action. In the remainder of Section Minimal Mechanisms and Coordination “Smoothers” in Joint Action and in subsequent sections, we will present how humans may also have a tendency to represent others' *value*, including affective-based outcomes (and expectancies) and how these may be brought to bear in Joint Action.

### The role of emotion in joint action

Vesper et al. (2010) has proposed a minimalist perspective on Joint Action, which emphasizes the sensorimotor coordination required in physical Joint Action tasks. They suggest that whilst classical *Joint Action perspectives* that address planning and high level reasoning are not well-equipped to deal with issues of fine-grained spatial-temporal sensorimotor coordination, the opposite is true of *sensorimotor-focused perspectives*. The focus of Vesper et al.'s has been to posit an approach for bridging the gap between these two perspectives by focusing on short-term planning, monitoring and predicting the actions of others. This minimalist approach views Joint Action as involving dedicated mechanisms for coordination and is concerned with *how* Joint Action is performed.

Much literature in Joint Action theory has concerned the shared representation of action effects (or outcomes), (e.g., Knoblich and Jordan, 2002; Sebanz and Knoblich, 2009). These minimalist approaches to Joint Action have, however, overlooked a potentially equally central aspect to Joint Action—*shared value states*, their expression, perception and inference. Where Joint Action is goal-based, representations of value provide a basis for expectations concerning the outcome of goal-directed behavior. By observing another's emotional state as an expression of anticipation of a goal-directed outcome or through contextually inferring its existence (e.g., empathizing), the monitoring burden (of other's actions and behavior) can be reduced.

Michael (2011), like Vesper et al. (2010), has advocated for a minimalist approach to the study of Joint Action, and suggested that emotions may have an important role to play in such an approach. Michael claimed “none of [the] minimalist proposals has addressed the potential role of emotions as coordinating factors in joint actions. In fact, no proposal of any kind has addressed this issue” (Michael, 2011, p. 3). However, there are indications that the potential role of affective^3^ states in Joint Action is beginning to garner interest. For example, the role of empathy, which, broadly, concerns the vicarious experience of particular affective states, has been alluded to in several recent Joint Action studies. It has been suggested that self-other representative states can only be understood in relation to the interdependence of motoric, cognitive and *affective* states (Sacheli et al., 2015; de Guzman et al., 2016; Milward and Sebanz, 2016; Steinbeis, 2016).

## An affective account of associative two-process theory

### Associative two-process

In this sub-section, we will discuss ATP theory (cf. Trapold, 1970; Urcuioli, 2005, 2013). We will also discuss differential outcomes training procedures that can illuminate a function for affective states in individuals. This description provides the foundation for understanding a minimalist affective learning mechanism (value function) for use in Joint Action.

ATP theory has been used to explain behavioral and learning phenomena that result when different (and arbitrary) stimulus-response (S-R) mappings are paired with different outcomes. These outcomes may be motivational stimuli, e.g., food pellets (for rewarding pigeons or rats), or they may be salient outcomes (e.g., light flashes, visual stimuli). The differential outcomes training paradigm has been used on non-human animals (typically rats and pigeons, cf. Peterson and Trapold, 1982), but also on infant and adult humans (e.g., Estévez et al., 2001, 2003; Holden and Overmier, 2014). According to this training paradigm, different outcomes are associated with different, but “correct”^4^, stimulus-response (S-R) mappings.

**Figure 1:**
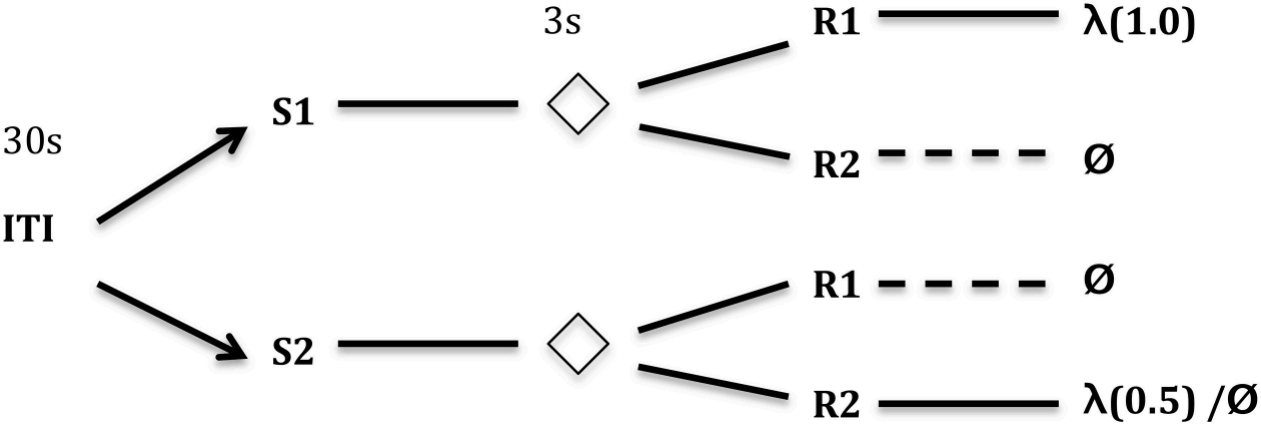
**Typical differential outcomes training schedule**. In this particular task, the training subject is required to respond differentially to one of two (or more) stimuli (S1, S2 in the figure) for every trial. After some delay (e.g., 3 s) where the Stimulus is removed, two (or more) new stimuli are presented which afford responses (R1 and R2 in the figure). Only one of the two responses gives a reward. Different S-R mappings, however, provide different outcomes (e.g., rewards). In the case depicted here, S1-R1 gives a reward 100% of the time, S2-R2 gives a reward 50% of the time—a differential outcome according to probability of reward (cf. Urcuioli, 1990). Other S-R mappings receive no reward. Key: ITI, inter-trial interval (in seconds); λ, reward probability; Ø, no reward.

**Figure 2:**
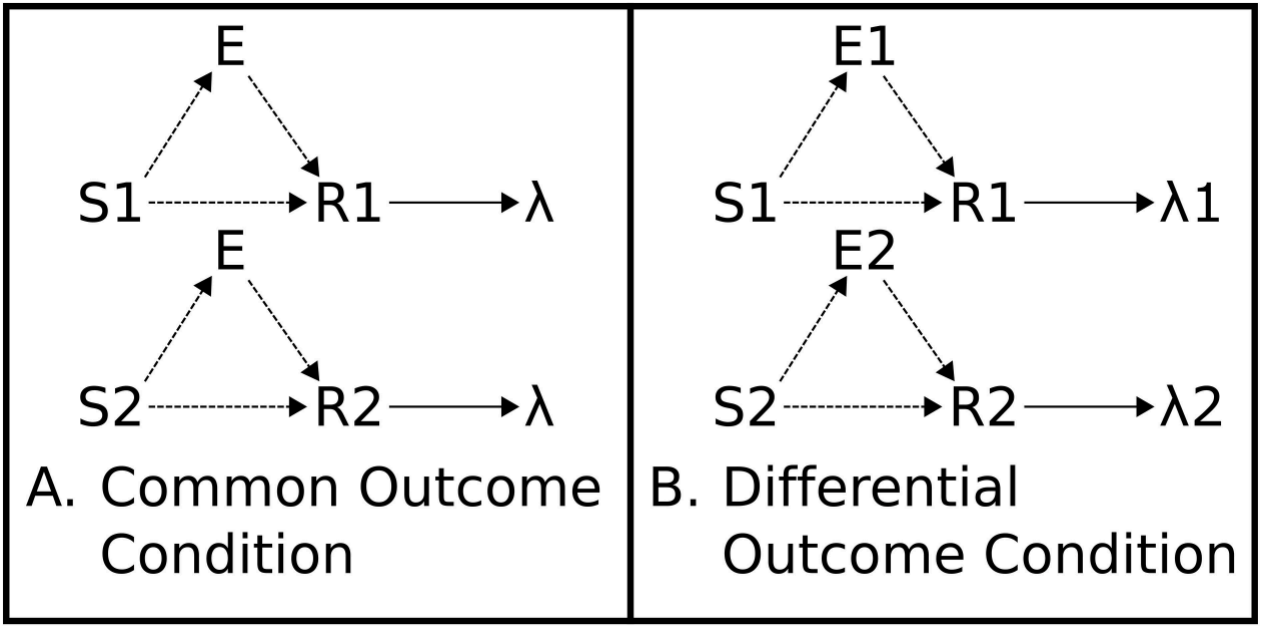
**Associative Two-Process Theory**. **(A)** Common Outcome Condition. Reinforced S-R associations (mappings) cannot be distinguished by outcome. **(B)** Differential Outcome Condition. Reinforced S-R associations can be distinguished, and cued, by differential outcome expectancies (E1, E2). Directional arrows indicate causal links. Dashed lines indicate learnable connections.

In the differential outcomes paradigm schematized in Figure 1, arbitrary task rules (S-R mappings) can also be learned but those “correct,” e.g., “rewarding,” mappings are associated with *differential* outcomes. In the example in Figure 2, the outcome may simply be the probability of reward (1.0 vs. 0.5) for making the correct response to the presented stimulus.

ATP theory proposes that outcome expectancies, during a training procedure wherein different S-R mappings lead to different outcomes, can cue responses in place of, or in combination with, the external stimuli. The outcome expectancy for a particular reinforcer becomes a stimulus: “the reinforcer itself is part of what is learned” (Urcuioli, 2005, p. 1). In this sense, the classical conception of the stimulus-response-outcome, or (S-R)-O, sequential relation (with S-R in brackets denoting that the stimulus-response association is learned), is more accurately portrayed as (S-E-R)-O where E is the learned expectation tied to a particular outcome. This relationship is captured in Figure 2, which shows how differential outcomes conditions yield different expectations in application of the different task rules (S-R mappings). These differential expectations provide, thereby, an additional source of information to response choice that can potentially facilitate, or even substitute for, the information about the task rules (S-R mappings).

**Figure 3:**
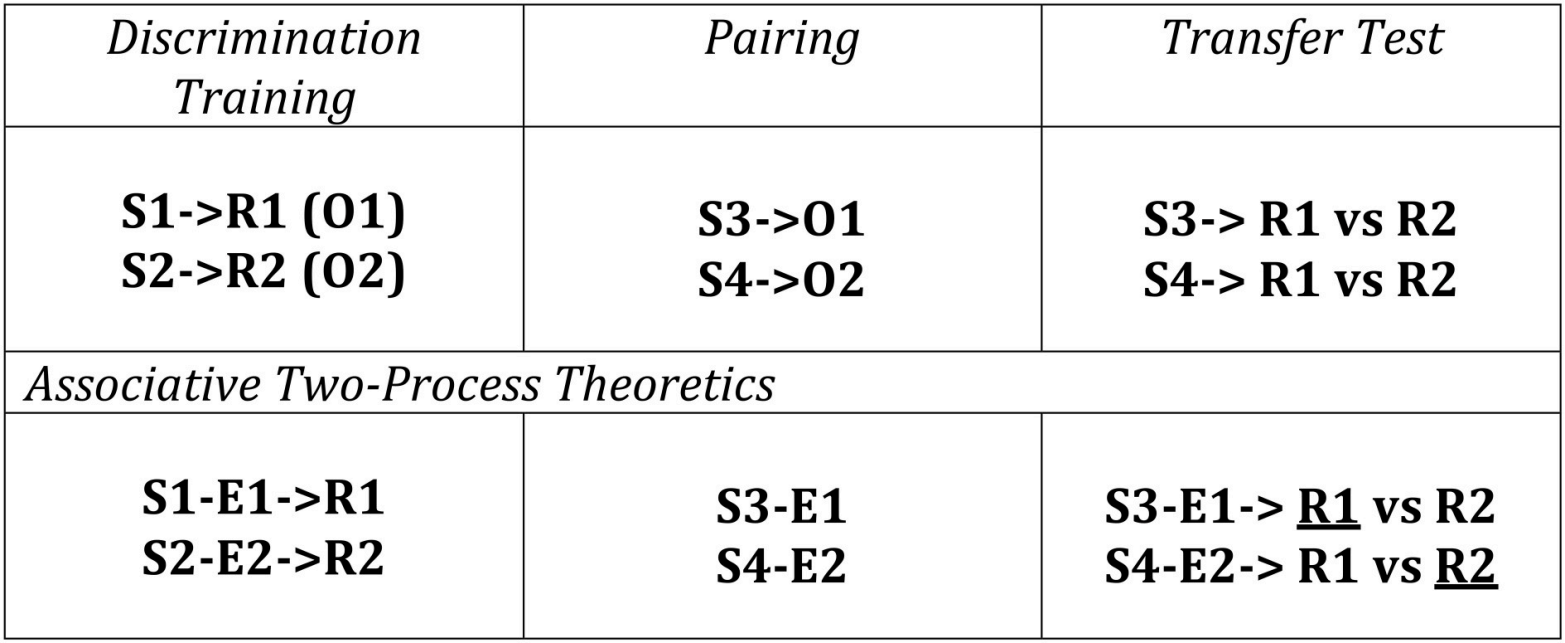
**Transfer of Control Paradigm with Differential Outcomes (Discriminative) Training**. The conditioning consists of three phases: Phase 1—a Discrimination Training phase where different stimulus-response (S-R) mappings (S1-R1, S2-R2) yield different outcomes (O1, O2); Phase 2—a Palovian learning phase where new Stimuli are presented and associated with previously experienced outcomes; Phase 3—an instrumental transfer phase where the Stimuli from Phase 2 are re-presented as are the response options from Phase 1. ATP theory predicts that responding will be based on already existing S-E and E-R associations learned from the first two Phases where the theorized preferred selections (underlined Rs) are shown here. This has been described in terms of cueing the response(s) associated with those stimuli classified by a common outcome—in this case S1 and S3 are classified by O1. Adapted from Urcuioli (2005).

Differential outcomes training procedures have also been applied to Transfer-of-Control (TOC) paradigms whereby learning and adaptive behavior is tested according to changes in the outcome contingencies that the individual experiences over learning trials. A schematic of a TOC is provided in Figure 3 along with the ATP theoretical explanation of the expected learning/behavior. The first two phases consist of a number of conditioning trials for the human / animal to make different associations based on S-R, S-E, and E-R contingencies. Since the outcomes (O1 and O2) are differential for the different S-R mappings in Phase 1 (Discrimination Training), it is possible to effectively *classify* new stimuli, introduced in Phase 2 (i.e., S3 and S4) by these same outcomes (cf. Urcuioli, 2005, 2013). As a result, when Phase 3 (Transfer Test) occurs, since the animal/human has learned to classify S1 and S3 according to the same outcome (O1)—that is, it has formed S1-E1 and S3-E1 associations—S3 automatically cues the response associated with E1 (learned in Phase 1). No new learning is required for this in spite of the fact that the subject has not been exposed to the task rule (S3-R1 mapping) previously. This transfer of control constitutes a form of adaptive switching.

Such a result cannot be explained by recourse to task rules (S-R mappings) alone. The S-E-R route (see Figure 2) provides the means for the subject to produce the adaptive response—it effectively generalizes its previous knowledge to the new setting. This S-E-R route is otherwise referred to as the prospective route (Urcuioli, 1990) since a growing expectation of an outcome is maintained in memory during the interval between Stimulus presentation and Response option presentation. This is contrasted to the S-R retrospective route so called as the memory of the stimulus is retroactively maintained in memory until response options are presented. Subjects can construct new task rules as a result of this type of inferential^5^ behavior.

### Associative two-process theory and affect

If we consider the schematized differential outcomes experimental set-up given in Figure 2, the different outcomes concern reward probabilities of 1.0 and 0.5, respectively. Overmier and Lawry (1979), and Kruse and Overmier (1982), suggested behavioral responding, following stimulus presentation, can be mediated by anticipatory frustration or reward according to the strength of the respective expectancies. In the sense of Figure 2, the expectancies (E1 and E2) can represent reward acquisition expectation, and reward omission expectation. Responses are associated with these two types of affective expectation as a function of how often they are rewarded. Thus, “anticipatory frustration … [can] gain at least partial control over one response, while the expectancy of reward [can gain] full control over the other” (Kruse and Overmier, 1982, p. 518). Kruse and Overmier (1982) provided evidence for this phenomenon experimentally. Whilst differential outcomes training procedures have focused primarily on differential sensory outcomes, or otherwise differences in magnitude of rewarding outcomes (and only sometimes on probabilities), the same principle may also be applied to differential punishing outcomes (Overmier and Lawry, 1979).

The notion of classifying emotionally-relevant stimuli by differential affective states has much in common with Damasio's (1994, 1999). Damage or absence (through lesioning) of brain structures (amygdala, prefrontal cortex) implicated in emotion elicitation and regulation led patients of Damasio into perseverative, overly rationalized or otherwise inappropriate decision-making. Damage to areas such as orbitofrontal cortex has also been implicated in rigidity of decision-making by Schoenbaum et al. (2003) (also Delamater, 2007) and Rolls (1999). In such cases, reversing responses to previously, but no longer, rewarded behaviors may be compromised.

Furthermore, Miceli and Castelfranchi (2014) have referred to a S->A->R mode of associative processing where A stands for “Affect.” In this case the links between affect and stimuli are hypothesized as being bi-directional (also see de Wit and Dickinson, 2009 for discussion). An adaptive benefit of this bi-directionality may be that affective states can have a role in selective attention of external stimuli suppressing attention to those stimuli incongruent with the present affective state.

### Affective associative two-process theory and joint action

To our knowledge, whilst differential outcomes training procedures and ATP theory have been applied to human learning and decision-making (cf. Maki et al., 1995; Estévez et al., 2001; Urcuioli, 2005; Esteban et al., 2014; Holden and Overmier, 2015), no application has been made to the social interaction domain. While the relevance of the paradigm—separate instrumental and pavlovian learning phases—might appear opaque to the types of Joint Action scenarios used to investigate the possibility of shared task representations given by Sebanz et al. (2005) and Atmaca et al. (2011), we suggest the significance of the above-mentioned Transfer of Control (TOC) paradigm to Joint Action is as follows:

1. Co-actors' observation of others' stimulus (event)—outcomes contingencies, permits a *type* of pavlovian learning.
2. Observing others' stimulus-outcome associations and learning therefrom, may help avoid the correspondence problem (mapping physical movements of others to those of self; cf. Brass and Heyes, 2005; Heyes and Bird, 2008) involved in learning by others' actions only.
3. Learning by differential outcomes can facilitate the learning of task rules both of self and other, as well as to lessen the importance of having *explicit knowledge* of task rules.

Although a *social* TOC paradigm does not directly entail Joint Action, similar to Sebanz et al. (2003, 2005), Atmaca et al. (2011), this paradigm may be used to provide evidence for tendencies for individuals to represent others' affective states *for use* in Joint Action. According to our postulates 1–3 above, being able to appraise events for self and emotionally appraise the state of the other serves as additional coordination facilitators that lessen the burden on monitoring and detecting the other's actions both in terms of learning how to perform a task and also in terms of learning the task (rules). Taking the example of Figure 3, one actor in the pavlovian (“Pairing”) phase would, instead of passively encountering newly presented stimuli, perceive these stimuli presented to an observed (co-actor). The observer would then vicariously experience, or otherwise learn, these associations and relate them to their own behavior. In this way, during the instrumental “Transfer Test” phase, the perceiver, having previously learned, for example, an E1-R1 association (“Discrimination Training” phase) and an S3-E1 association (vicariously in the “Pairing” phase), would in the “Transfer Test” phase already have access to the S3-E1-R1 affective (anticipatory) route that can substitute for explicit knowledge of (or exposure to) the S3-R1 task rule. This means that without having to learn, the observer would be able to transfer vicariously experienced knowledge to his/her own behavior.

Consistent with the requirement of minimal monitoring for spatiotemporally synchronized Joint Actions (Vesper et al., 2010; Michael, 2011), the requirement for the above-described social transfer of control (or social TOC) would be that the observer is, minimally, attentive to the co-actor's stimuli and outcomes but would not require monitoring of ongoing actions. Requisite to this perspective are neural-computational mechanisms that can relate other's outcome, or expected outcome, to one's own response repertoire. We will turn to this in the next section.

## Neural-computational basis for affective valuation

### Neural-computational basis for affective valuation in individual action

In previous work we have described a computational model of differential outcomes expectancies based on reward (acquisition) expectation and reward omission expectation learning (Lowe et al., 2014). Our model provided a qualitative replication, in simulation, of the results of Maki et al. (1995) and Estévez et al. (2001) concerning differential outcomes training of infants of different ages between 4 and 8.5 years of age. We describe here only the expectation-based component of the model responsible for learning S-E associations. This component of the model is focused on due to the role it plays in affectively “classifying” stimuli permitting transfer of control. It thereby provides the basis for the prospective route of behavior. The full model is found in Lowe et al. (2014).

**Figure 4:**
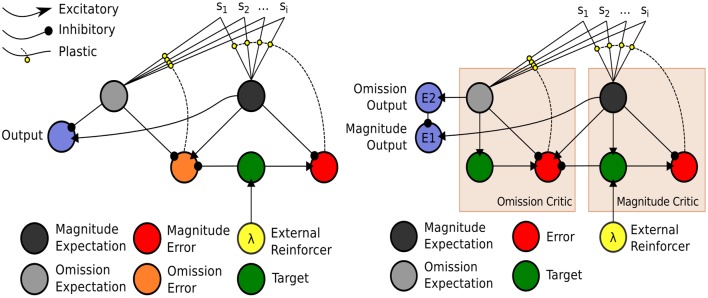
**Computational Models of Differential Affective States**. **Left:** Neural Network based computational model of Reinforcer Magnitude and Omission Learning of Balkenius and Morén (2001). **Right:** Temporal difference learning neural network adaptation of Balkenius and Morén (2001) given by Lowe et al. (2014).

The model, depicted in Figure 4 (right), is a temporal difference (TD) learning neural network instantiation of the Balkenius and Morén (2001) network (Figure 4, left). This TD network, contrary to standard TD learning algorithms computes a value function according to two dimensions: *magnitude*, or reward strength, and *omission*, or reward omission probability. Specifically, the value function computes temporally discounted reinforcer (reward or punisher^6^) magnitude (right-side of network) valuation of a given external stimulus (S1, S2,…Si) presented to the network. From this magnitude valuation is derived an omission valuation. Although, Balkenius and Morén (2001) did not explicitly state that the “omission” node (depicted in our network schematic of the model) computes omission *probability*, it effectively does so as a fraction of the magnitude size; therefore, given that the reinforcer magnitude presented to the network is equal to 1.0, the omission valuation will be a probability computation based on experience. The requirement for omission computation is that the magnitude network learns, but does not unlearn, the valuation of the reinforcer. The omission network, on the other hand, necessarily both learns and unlearns its valuation using prediction errors so as to refine its omission probability approximation. This functionality is correspondent to the orbitofrontal cortex (cf. Schoenbaum et al., 1998, 2003; Delamater, 2007; Watanabe et al., 2007). The requirement for the magnitude network to not unlearn is biologically plausible when this part of the network is considered to implement the learning in the amygdala. Morén (2002), for example, states: “There are studies that imply that conditioning in the [a]mygdala is permanent, or at least very hard to reverse … The rationale behind this is that once learned, a reaction—especially a negative one—is so expensive to retest that it pays to assume this negative association is valid everywhere unless definite proof otherwise has been established” (Morén, 2002, p. 85). The network, thus, does not unlearn the value but through inhibition of the output of the reinforcer magnitude network, can learn to inhibit behavioral responding. The model has been demonstrated to capture the “savings effect” whereby behavioral responding, inhibited as a result of a reinforcer no longer being presented to the network, is relearned more quickly than it was initially acquired. This occurs as a result of the relatively fast learning rate of the omission network in the model. This implements a known computational component of the orbitofrontal cortex, i.e., fast and flexible “reversal” learning (cf. Schoenbaum et al., 2007). Our temporal difference learning adaptation of this model (Figure 4, right-side) addresses one limitation of the Balkenius and Morén (2001) model, that is lack of sensitivity to the interval between stimulus presentation and reinforcer onset. Our model thereby implements a “prospective” component of learning—temporal difference based valuation. The TD learning model of Sutton and Barto (1998) predicted the profile of dopaminergic phasic activation transfer from reinforcer onset to earliest predictive stimulus onset (Schultz, 1998, 2007). The equations of our model are given in the Appendix section (Appendix A). They adapt Doya's (2000) continuous time TD learning equations providing a more neurobiologically plausible implementation of Sutton and Barto's (1998) discrete time TD learning. The TD learning mechanism in our model is described and exemplified in detail in Appendix B.

The outputs of the two value functions (for magnitude and omission), when non-linearly transformed, e.g., by a sigmoid function, serve to approximately *classify* outputs of this value computational network. In the example in Figure 4, non-linearly transformed output (of 0.5) will provide strong inhibition to the output of the Magnitude value function (see Figure 4, right). This stimulus (stimulus 1), is thus classified by omission expectation—a *pessimistic* affective judgment—and its output may be associated with particular responses (permitting the E-R associations of ATP theory). Conversely, a low omission probability is insufficient to inhibit the magnitude output leading to a dominant *optimistic* affective judgment.

In the Balkenius and Morén (2001) model (Figure 4, left), outputs of both the omission and magnitude value functions are linear. It should be noted that using a heaviside threshold function allows for complete classification but at the expense of failing to generate the faster (re)learning characteristic of the savings effect that the Balkenius and Morén (2001) model captures. Thus, the output functions in our model, in using semi-linear functions are able to produce both approximate affective (pessimistic/optimistic) classifications of stimuli whilst preserving the savings effect.

The manner in which stimuli classified by differential outcomes can then be associated with responses, consistent with biologically plausible TD learning methods, e.g., Actor-Critic methods (cf. Houk et al., 1995), concerns use of a three-factor learning rule. This is hebbian learning (pre-synaptic and post-synaptic activations are associated) gated by the reward prediction error generated by the “Critic,” which in our model is the inverted prediction error produced by the Omission “Critic” (also see Lowe et al., 2014). ATP theory has been used to explain generic differential outcomes learning findings (Urcuioli, 2005). However, as described with recourse to our neural-computational model, a *type* of differential outcomes learning involves classifying stimuli by differential probability of reward (cf. Overmier and Lawry, 1979; Kruse and Overmier, 1982). Where probabilities are sufficiently distinct, differential expectations are learned that concern an expectation of an omission of reward and an expectation of an acquisition of reward. A network that implements expectation-based learning of this type can be likened to Rolls (1999, 2013) stimulus-reinforcer contingency “appraisal” model. The neurobiological underpinnings of this network Rolls considered to be the orbitofrontal cortex (OFC) as it interacts with the amygdala. Interestingly, Watanabe et al. (2007), in relation to work by Hikosaka and Watanabe (2000, 2004), described the finding of neural activity in the orbitofrontal cortex correlating with omission of expected reward during a delay period (from predictive cue onset to the time at which reward is intermittently delivered). McDannald et al. (2005) have suggested that it is the interaction between the orbitofrontal cortex and the basolateral component of the amygdala (BLA) that is responsible for the encoding of reward and omission expectations associated with eliciting primary stimuli and responses: “the OFC and the BLA form a circuit that may mediate both learned motivational functions and the use of outcome expectancies to guide behavior” (p. 4626). Delamater (2007) has, similar to McDannald et al. noted impairments in differential outcomes-based and devaluation (omission)-based learning as a result of OFC lesions. Concerning links between Stimulus valuations (i.e., S-E associations) and how they bring to bear on decision making (i.e., via E-R associations), medial prefrontal cortex (Passingham and Wise, 2012), and dorsolateral prefrontal cortex (Watanabe et al., 2007) have been suggested to have respective roles in outcome-contingent choice, and integration of retrospective and prospective memory that may provide a sort of competition mediating response choice.

In sum, there exists abundant neurological and behavioral evidence for this neural computational model of ATP theory providing an affective value function.

### Neural-computational basis for affective valuation in joint action

In the domain of Social Neuroscience, which dates back to Cacioppo and Berntson (1992), a key controversy to the present day, and critically significant to Joint Action, concerns whether social value qualitatively differs from non-social value or is fundamentally the same but entails differential pre-processing of (social) stimuli. Resolving this debate is central to understanding the extent to which individuals can detect and monitor the affective states (expected outcomes) of others for facilitating Joint Action.

Adolphs (2010) discussed whether social processing is unique or whether the information processing is of the “same type” as non-social processing. He categorized social processing into: (i) *social perception*, (ii) *social cognition*, (iii) *social regulation*. Of the three domains of information processing identified, all are related to the processing of affective information. Adolphs, further stated: “An important question for the future is therefore why, when, and how emotion participates in social cognition” (p. 755).

#### Social valuation: extended common currency (ECC) vs. social-valuation-specific (SVS)

Ruff and Fehr (2014) reviewed whether a neurobiological distinction between *social* and *non-social* value can be made. They highlighted three core aspects of value: (i) Experienced value, (ii) Anticipated value, (iii) Decision value. In the case of (i), orbitofrontal cortex (OFC), amygdala, insula and anterior cingulate cortex (ACC) are linked to the experience of actual reward (or punishment). In the case of (ii), value concerns the use of prediction errors as they derive from anticipated-value signals. In individual decision-making, dopaminergic neurons encode prediction error signals while striatum, OFC and amygdala are said to constitute the reward neural circuitry correlating with value anticipation (cf. Schoenbaum et al., 2003, 2007; Rolls, 1999). Decision value (iii), on the other hand, concerns choice-based preference and is differentiated from anticipated reward value. Its strongest neural correlate, according to Ruff and Fehr (2014), appears to be in the ventral medial prefrontal cortex (vmPFC)—also see Damasio (1994, 1999).

**Figure 5:**
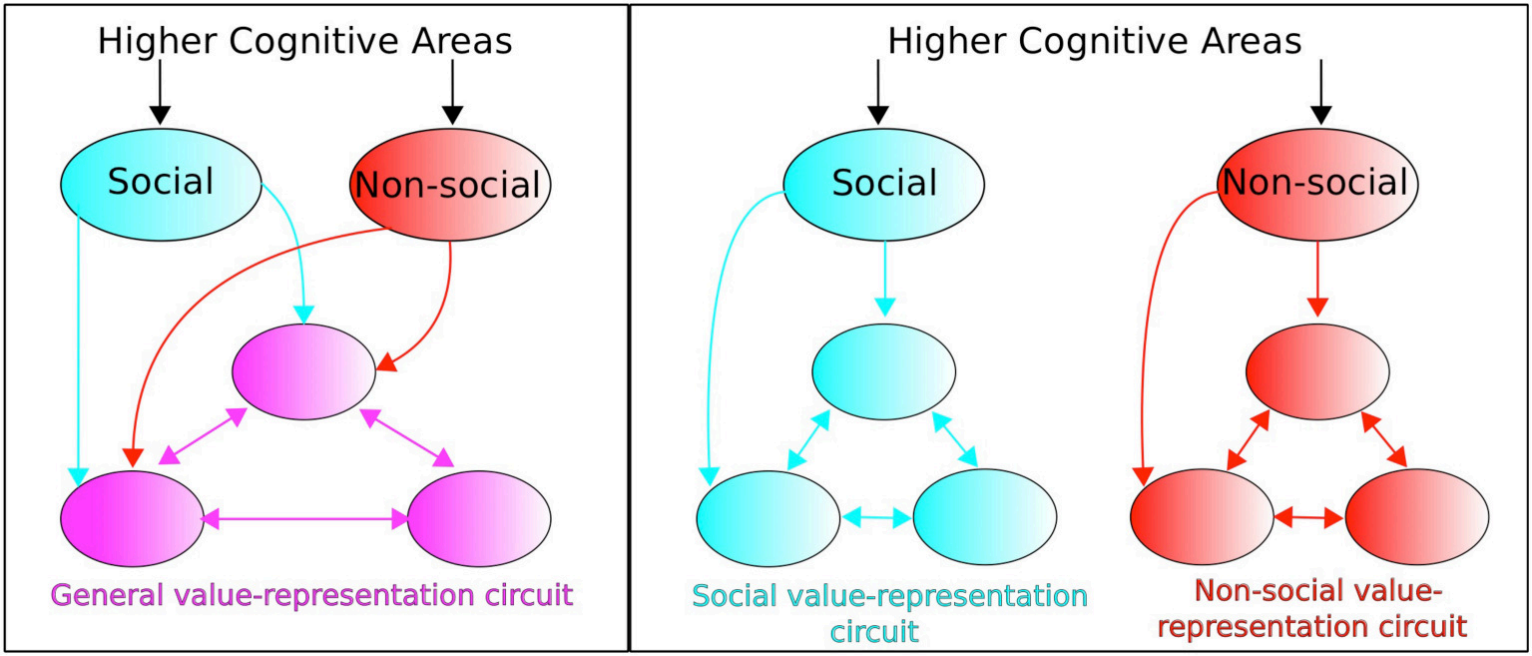
**Extended common currency (ECC) vs. social-valuation-specific (SVS) circuitry**. The two perspectives entail: (**Left**; ECC) processing of social and non-social stimuli using the same value-representation circuitry, vs. (**Right**; SVS) processing of social and non-social stimuli but within dedicated non-social and social-valuation specific circuitry. Adapted from Ruff and Fehr (2014).

The above value components have been considered within a social value conceptual framework. Ruff and Fehr identify a dichotomous perspective in the empirical and modeling literature regarding neural circuitry concerned with valuating social signals. On the one hand, social value representations are considered as utilizing the neural circuitry of non-social value representations (“identical neural processes assign motivational relevance to social and non-social factors,” Ruff and Fehr, 2014, p. 550). This constitutes an “extended common currency” (ECC) perspective whereby distinction between social and non-social information is made outside the value-representation circuit (see Figure 5, left). An alternative perspective concerns social value and non-social value being represented in separate dedicated circuits (see Figure 5, right) whose anatomical structure and computational processing may, nevertheless, be similar or even identical.

The particular set of modules comprising the value representation are considered by Ruff and Fehr (2014) to “not show specific brain areas and connections but rather…abstract principles of how brain areas and their interactions could implement these computations,” (Ruff and Fehr, 2014, p. 551). Such areas can include, therefore, value components that concern (i) Experience, (ii) Anticipation, (iii) Decision, valuation, as listed above. Whether all three aspects of valuation should be considered to fall into the ECC or SVS perspective is not addressed by Ruff and Fehr (2014), however.

#### Social valuation and joint action

**Figure 6:**
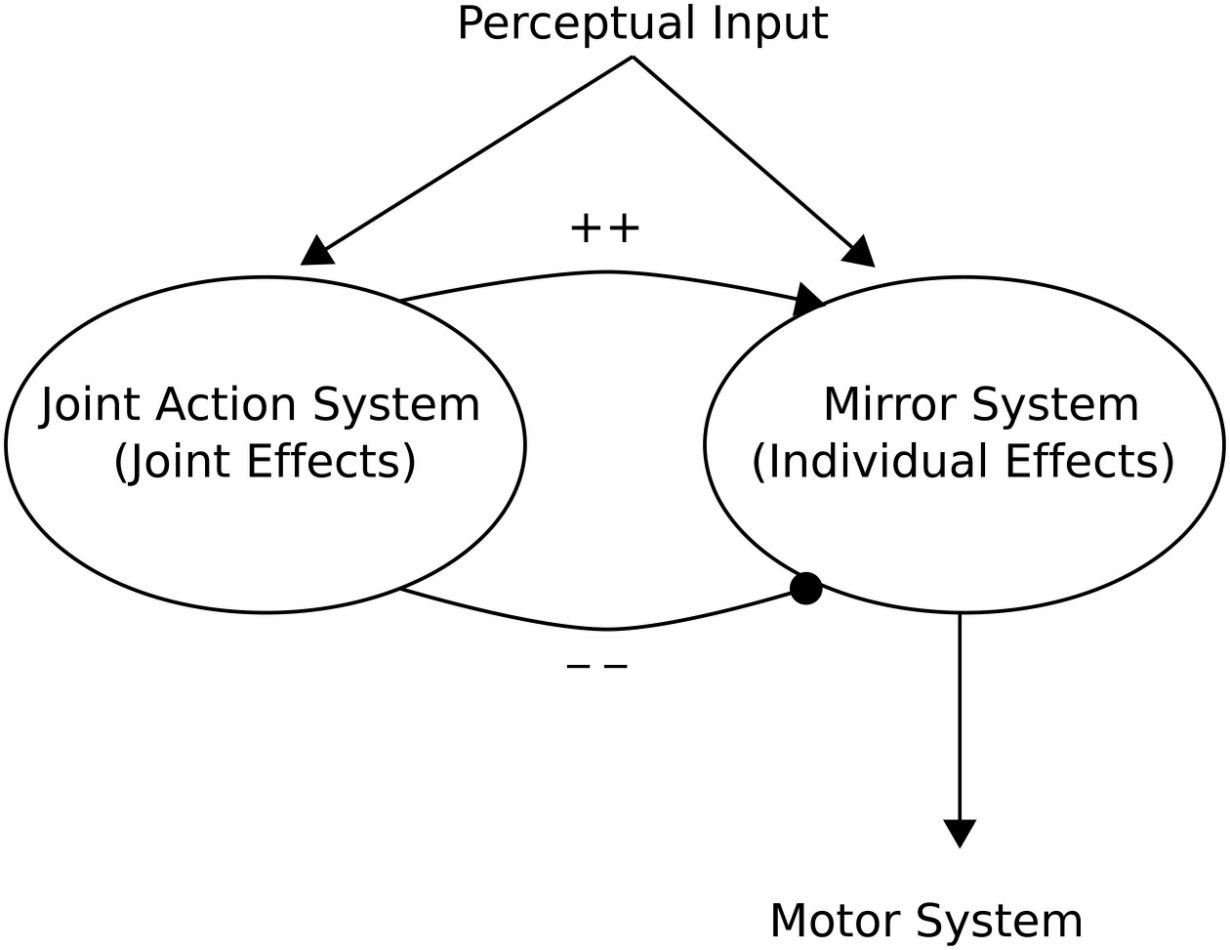
**Knoblich and Jordan (2002) Joint Action schema**. The schema consists of two main aspects: (1) A Mirror (neuron) System whose activity may reflect either the individual effects of the “Self” or those of a perceived “Other”; (2) A Joint Action System whose activity reflects the action outcome effects of Joint Action. Adapted from Knoblich and Jordan (2002).

Knoblich and Jordan (2002) provided a high-level “minimalist” Joint Action Architecture based on action outcome effects of a mirror neuron system (see Figure 6). This can be seen as providing a framework from which to interpret models pertinent to Joint Action. In this architecture, a mirror neuron system becomes active when *either* the individual registers outcomes of actions (e.g., the expected end point of an action), *or* when the individual observes another organism achieving the same action outcome. This implies an ECC hypothesis as advanced by Ruff and Fehr (2014).

In this Joint Action context, however, these “social” and “non-social” effects are further modulated by a system that accounts for the complementarity of an individual or other's action. Thus, if the particular task requires Joint Action and the engagement with other is perceived as such Joint Action, the actions of self and other may be modified. Bicho et al. (2011), produced a neural-(dynamic) computational architecture of Joint Action that implements such a division between joint action, and individual components for use in an autonomous robot that was able to interact, via dialogue, with humans according to a task that required complementary actions.

While neural computational architectures of Joint Action and *emotions* exist (cf. Silva et al., in press)^7^, we are not aware of those that focus on affective learning mechanisms that comprise TD-based value functions. Suzuki et al. (2012) identified “[a] fundamental challenge in social cognition [*which is*] how humans learn another person's value to predict [*their*] decision-making behavior” (p. 1125). Another important question from the perspective of the nature of social value functions concerns: how humans learn another person's value to inform their *own* decision-making behavior. These two issues allude to Ruff and Fehr's (2014) identification of Anticipatory, and Decision, value where a separation may be made between valuation of stimuli (Anticipatory) and valuation of choices (Decision).

**Figure 7:**
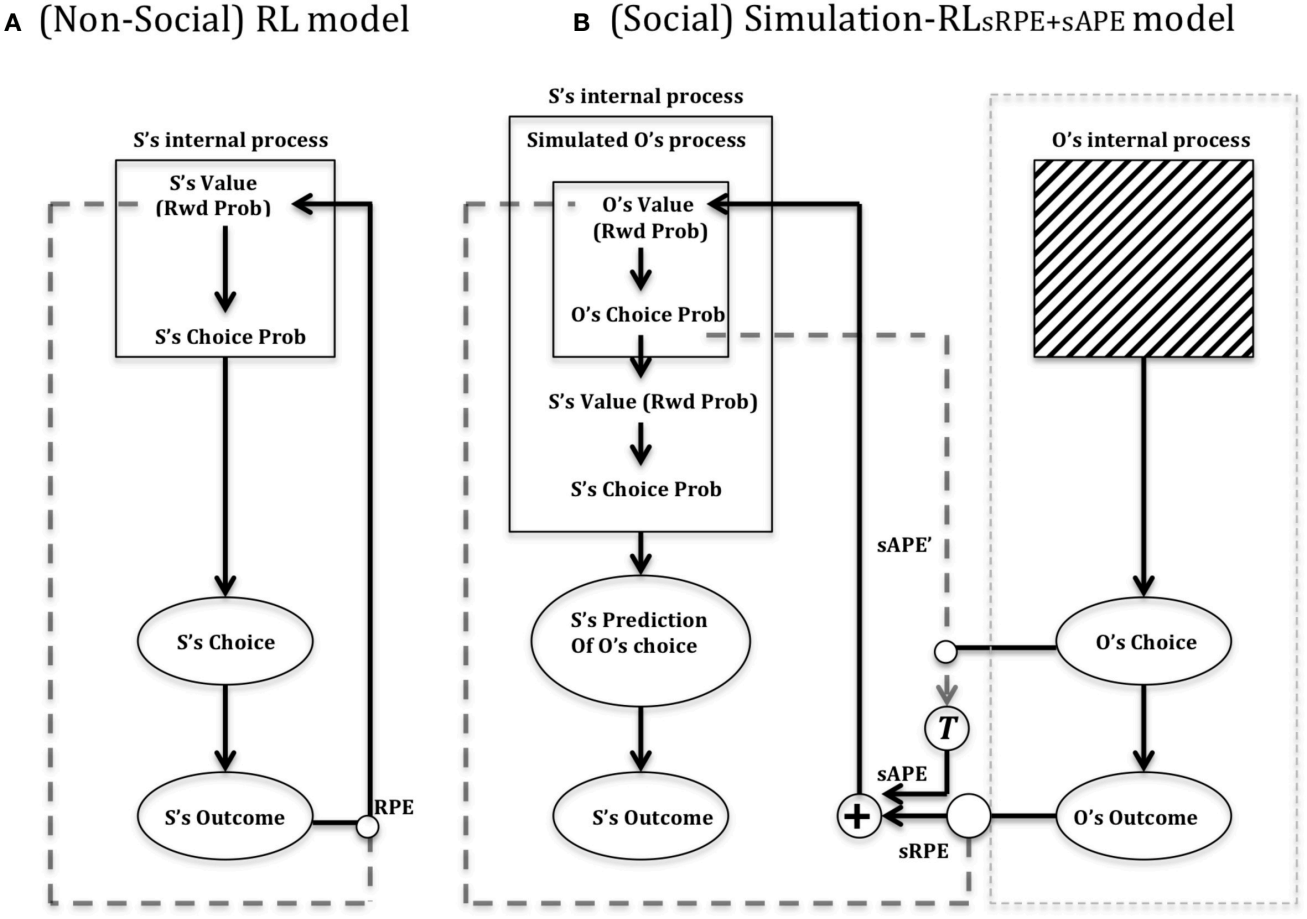
**Suzuki et al. (2012) reinforcement learning model of social value**. **(A)** RL model: Suzuki et al. (2012) provide a depiction of a standard reinforcement learning circuit, which (as for our model shown in Figure 4), updates a value function (reward probability) according to a reward prediction error (RPE) that compares the reinforcement (reward) outcome (S's Outcome) to the expected value (Rwd Prob), following a particular behavioral choice. The Choice probability is based on a stochastic action selection process that compares the different action options based on their previously experienced/learned probability of yielding reward. **(B)** Simulation-RL model. Central to this model is the use of simulated prediction errors by the Self (S) of the Other (O) to update a predicted value function of the other. The model assumes that the Other's internal process (actual value) is a black box whilst action choice and outcome of other are perceptible. See text for main details. Key: sAPE, simulated action prediction error; sRPE, simulated reward prediction error; RPE, (Self) reward prediction error; T, transformation function of sAPE into a value usable for updating the Other's value function. Adapted from Suzuki et al. (2012).

In Figure 7 is depicted Suzuki et al.'s (2012) reinforcement learning model of social value. In Figure 7A (left) is shown a standard (non-TD) Reinforcement Learning (RL) model that updates a value function for the self (S) based on the reward prediction error (RPE) generated following action selection. Each action is valuated by previous experience according to whether it leads to reward or not. In this model, unlike our model (illustrated in Figure 4, right), a single value dimension is depicted which is labeled “Rwd Prob” (i.e., reward probability). Reward magnitude, held constant in the social condition of Suzuki et al. (2012), was multiplied by reward probability. Figure 7B (right) shows Suzuki et al. (2012) Simulation-RL model. Like others in the field (cf. Behrens et al., 2007; Burke et al., 2010), Suzuki et al. (2012) posit the existence of two types of *simulated* prediction error that can be used when predicting the outcome of the *Other* in a particular task. An sRPE (simulated reward prediction error) uses the perceived outcome of the Other to update a predicted value function of the Other. Replicating the *Self* value function (Figure 7, left), this function valuates different actions, which are then compared as part of action selection. Moreover, the use of sAPE (simulated action prediction error) updates the Other's value function, which is used to help predict the choice of the Other increasing the ability to predict the Other's outcome and subsequent response choice.

In the validation experiment of Suzuki et al. (2012), they found that their Simulation-RL model was better able to capture behavioral data of participants in a condition requiring them to predict the choices of another subject (in reality a computer program). These choices were valuated by an abstract and probabilistic monetary reward. The Simulation-RL model replicated the empirical data relatively worse, though still fairly accurately, when only sRPE was used as compared to both sRPE and sAPE (reward and action prediction errors). The model did not match the empirical data at all when using only the (Self) RPE or only the sAPE.

Suzuki et al. (2012) found that reward prediction error (and simulated reward prediction error) was correlated with neural activity (BOLD signals) in the ventral-medial prefrontal cortex (vmPFC) indicating that, consistent with the ECC perspective of Ruff and Fehr (2014), the simulation of Other's outcome prediction errors recruits circuitry used for individual outcome prediction errors. The authors suggested that their findings provided: “the first direct evidence that vmPFC is the area in which representations of reward prediction error are shared between the self and the simulated-other,” (Suzuki et al., 2012, p. 1132). More generally throughout the decision making process made by Self (for Self) and Self on behalf of Other, vmPFC showed very similar activation in both cases: “the same region of the vmPFC contains neural signals for the subjects' decisions in both the Control and Other tasks, as well as signals for learning from reward prediction errors either with or without simulation,” (Suzuki et al., 2012, p. 1132). This finding would suggest that at least one component of value identified by Ruff and Fehr (2014), i.e., Anticipatory value, is shared in neural-computation of value of Self and of Other.

On the other hand, dorsal lateral/medial prefrontal cortex was implicated in generating a simulated action prediction error (of Other). Ruff and Fehr (2014) interpreted these findings as being evidence of a Social-Valuation-Specific (SVS)—see Figure 5 (right)—explanation of social stimuli processing based on “spatially and functionally distinct prediction errors that nevertheless follow similar computational principles” (p. 556).

In relation to the Joint Action architecture of Knoblich and Jordan (2002; Figure 6), the Suzuki et al. (2012) architecture (Figure 7, right) embeds within an individual circuit additional computational processes for simulating the (action and outcome) effects on other that then lead to motoric outputs in the self. Simulated other prediction errors (correlating with vmPFC activity) provide a basis for a “shared representation” of value that may be requisite to coordinated joint activity (e.g., Joint Action).

#### Social valuation and ATP

Let us now refer back to Section Associative Two-Process and the traditional use of TOC experiments as a means of validating the existence of an ATP (See Figure 3). Pavlovian conditioning, as a passive form of learning, i.e., where the subject's responses do not influence the onset of stimuli and outcomes, may also be conceived in a social context. In relation to the pavlovian phase in Figure 3, we postulate that individuals, rather than passively perceiving Stimulus-Outcome pairs in relation to *Self*, may perceive Stimulus-Outcome pairs in relation to *Other*. In the sense of the Suzuki et al. (2012) model/experiment described in Section Social Valuation and Joint Action, the subject may perceive the Other's observed (reward) outcome. This could be the result of at least three experimentally manipulated interaction scenarios: (i) *Competitive*—the Other receives a non-reward (or punisher); (ii) *Collaborative*—the Other receives a reward (that benefits Self); (iii) *Vicarious*—the Other receives a reward (neutral to the Self). Suzuki et al.'s (2012) set up explicitly concerned scenario (iii) here. In their set-up external reward was, however, provided for correctly predicting the other's choice (vicarious decision making). The authors provided behavioral and neural-computational modeling evidence to suggest that vicarious reward was not merely ego-centrically experienced, i.e., where the other's actions and outcomes were not perceived as belonging to the other.

The individual's knowledge of the social interaction scenario in which (s)he is placed permits differential pre-processing of social stimuli thereafter valuated according to ECC or SVS neural computational circuitry. Such pre-processing involves perceiving Other as competitor requiring a comparing of outcomes (i), or as a collaborator requiring monitoring of collectively attained outcomes (ii), or focus purely on Other's outcomes (iii).

Central to the perspective of ATP theory is that individuals are able to transfer knowledge (and “generalize,” or *best guess*) from previously experienced instrumental and pavlovian phases to a novel instrumental phase, i.e., one in which *new* Stimulus-Response (S-R) pairings are presented. Using the prospective (Stimulus-[Outcome]Expectation-Response) route, given a differential outcomes and transfer-of-contrl set-up, subjects are able to, with minimal or no learning, find the correct S-R mapping. Given *Collaborative* or *Vicarious* rewarding social scenarios as outlined above, independently representing outcomes of Other subjects is less obviously “typical” (does not require comparison of Self to Other) than for *Competitive* scenarios (does require comparison).

We consider a social version of the TOC using a differential outcomes procedure an excellent methodological paradigm for testing an ECC hypothesis, specifically, our Social Aff-ATP hypothesis. This is because if the individual case of transfer can also apply to the social case (transfer based on simulated Other's Stimulus-Outcome pairings), subjects can potentially substitute for a lack of information (about action choice). They can do this through: (i) the use of observed differential outcomes (values) that they vicariously experience or/and (ii) the perception of expressions of Other that map to the Other's value functions (outcome expectations/predictions). Such adaptive switching behavior may facilitate coordination of Joint Action, i.e., provide a coordination *smoother* (Vesper et al., 2010).

#### A neural computational implementation of social Aff-ATP

**Figure 8:**
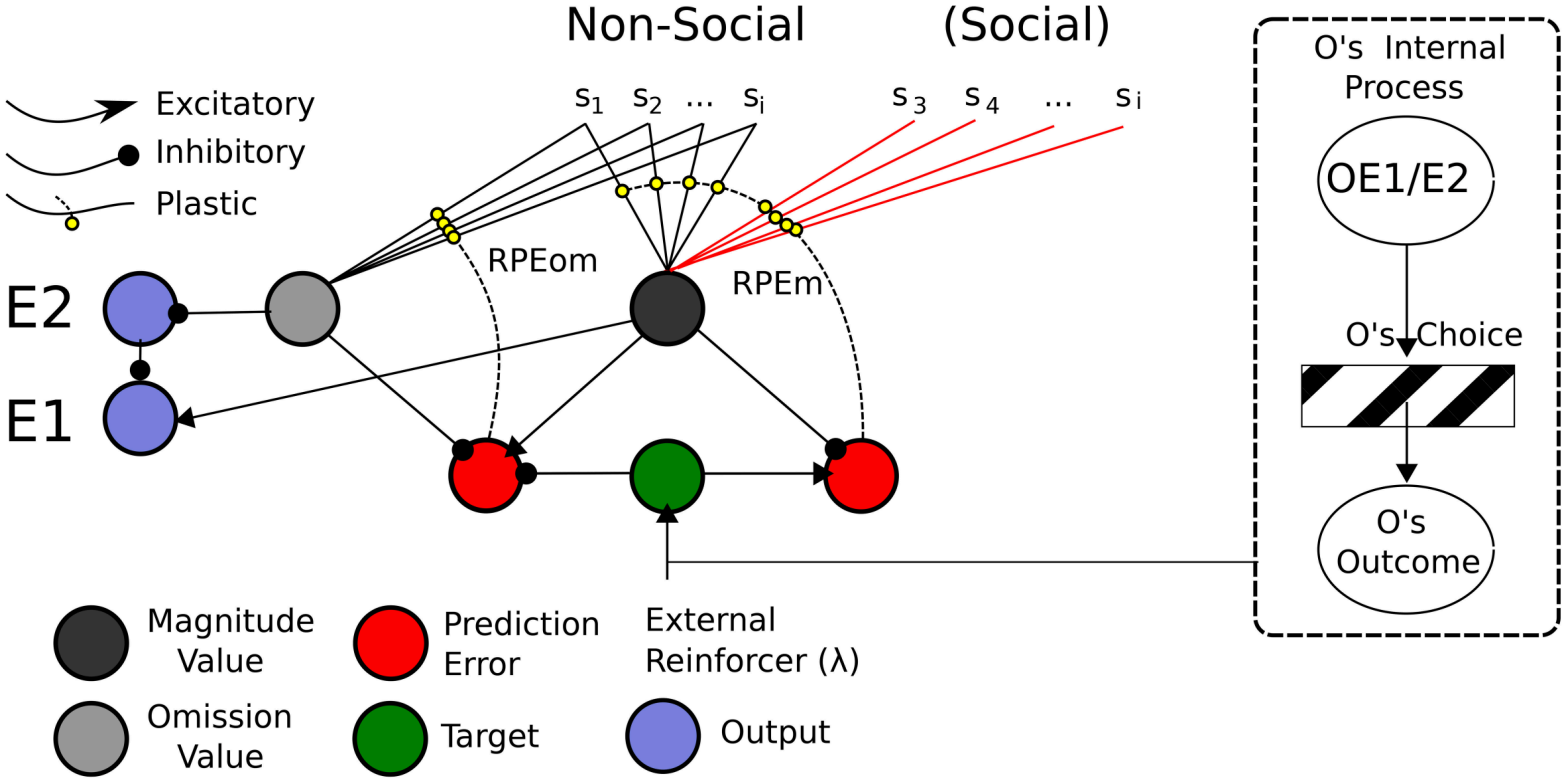
**ATP Neural Network required for Social TOC**. Adapting the Balkenius and Morén (2001) network, only the stimuli used in the social Pavlovian phase are added. These “Social” stimuli would be required to have direct input into the Non-Social value function. In this sense, social valuations would directly recruit the existing non-social network. This does not preclude the existence of other social valuation representations (e.g., of the like depicted in Figure 9), however. On the right hand side is shown “Other” inputs to the network. It may be possible that the Other's internal process is not so opaque given that affective expressions (of expectations or outcome reactions) map to affective states isomorphic to those of the observer's.

In this sub-section, we present a *Social Aff-ATP* neural computational mechanism that constitutes our hypothesis in this article. It is depicted in Figure 8.

The mechanism parsimoniously adapts that of Balkenius and Morén (2001), Balkenius et al. (2009) and Lowe et al. (2014)—see Figure 4—by viewing social processing as a pre-valuation stage (not described here) that exploits the existing function for individual/Self stimuli valuations. The neurobiological, behavioral and neural-computational evidence for the existence of this mechanism, previously considered in terms of individual effects, was given in Section Neural-Computational Basis for Affective Valuation in Individual Action. It is an explicit implementation of the ECC schema of Ruff and Fehr (2014). It also comprises a type of mirror system as described by Knoblich and Jordan (2002) but as it applies to perception/representation of (affective) value outcomes rather than action effects. We discuss below how this mechanism, while not accounting as comprehensively for Joint Action effects as the full Knoblich and Jordan model, permits effects that can be *used for* Joint Action and is thereby more minimalist. The equations for this mechanism, in viewing social inputs as stimuli to be valuated, are the same as for the originally-conceived individual (non-social) model found in Appendix A.

This mechanism is apt for processing social valuations in *vicarious* and *collaborative* social scenarios where representations of Other are not functionally critical.

**Figure 9:**
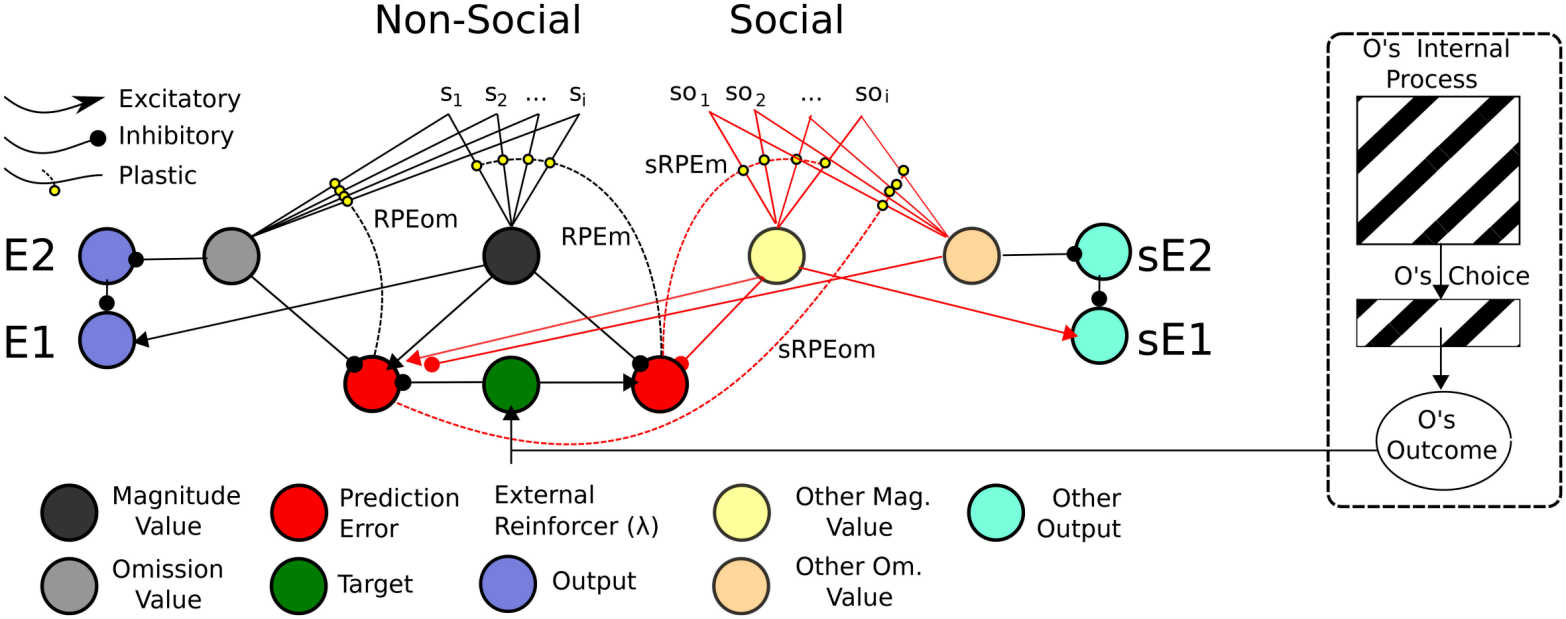
**ATP Pavlovian adaptation of Suzuki et al. (2012)**. The Non-Social network used the output function of Lowe et al. (2014) and the value function of Balkenius and Morén's (2001)—we use this latter function rather than our own temporal difference learning version to simplify the illustration. As for Suzuki et al. (2012), the prediction error nodes for Magnitude and Omission expectations are used both by the Non-Social network to update the Self's value function, but also by the social-valuation-specific network. Simulated Other (Social) value predictions (for Magnitude and Omission), as for Suzuki et al. (2012) are separately represented and have separate outputs. The Other's internal process here may still be seen as a black box as is the Other's choice. The only information required is the Other's Outcome.

An alternative mechanism to our Social Aff-ATP is provided in Figure 9. Here valuation of social stimuli is computed using a separate “Other” /Social value circuit and is considered a SVS circuit. More specifically it is a SVS-ATP alternative mechanism.

Consistent with Suzuki et al. there are separate Non-Social and Social value functions for Self and (simulated) Other. Also consistent with Suzuki et al. (2012), both Non-Social and Social value functions recruit the same reward prediction error computations. Suzuki et al. suggest these computations correlate with vmPFC (BOLD) neural activity. Such prediction errors have also been attributed to activity in the striatum (cf. Redgrave et al., 1999). Our interest is, above all, in the neural computational plausibility of such a network rather than the specific anatomical root of the computations. However, in line with Balkenius and Morén (2001), as mentioned in Section Associative Two-Process Theory and Affect, we posit that the value nodes compute activity correspondent to that in the orbitofrontal cortex and amygdala^8^. In our ATP model, prediction errors code for reward magnitude (a non-negative value) and reward omission, i.e., two dimensions of value whose outputs relate to emotions elicited according to the acquisition and omission reward contingencies, respectively (cf. Rolls, 1999).

The depicted mechanism in Figure 9, as for the Social Aff-ATP mechanism (Figure 8), outputs value computations of magnitude and omission to relay nodes (E2 and E1) whose function is to non-linearly “classify” the outputs of the stimulus valuations as omission expectant (pessimistic) or acquisition expectant (optimistic)—see Lowe et al. (2014) for computational details. These outputs in turn, through a three-factor learning rule (hebbian learning gated by reward prediction error/dopaminergic projections) can be associated with actions / choices. In the alternative mechanism shown in Figure 9, the Social value representation projects instead to sE1 and sE2 relay nodes, i.e., separate output representations. For the Social Aff-ATP (and SVS-ATP) mechanism we do not focus on the action selection component of the algorithm, which can be represented simply by a winner-take-all laterally inhibiting layer of nodes (each node representing an action/choice). Nevertheless, mathematically, the link between value function output and action selection in Suzuki et al. and the Social Aff-ATP mechanism are analogous. For Suzuki et al. stimulus valuations are computed as: *Q*(S1) = *p*(S1)*R*(S1), where *Q*(S1) is the valuation of stimulus 1 (S1) computed as the product of probability of reward for S1, i.e., *p*(S1) and magnitude of reward for S1, i.e., *R*(S1). In the Social Aff-ATP (and SVS-ATP value functions), E1 is calculated as E1 ≈ *R*(S1) − (1 − *p*(S1)), where (1 − *p*(S1)) = omission probability and is given by the relayed output of E2 subject to non-linear transformation. When *R*(S1) is fixed at 1.0, as it is for Suzuki et al. in their social condition, E1 = *Q*(S1). A difference in our ATP-based models is that both pessimistic/omission probability focused (E2) and optimistic/acquisition probability focused (E1) outputs are permissible allowing for differential expectation-response associations. Another difference is that Suzuki et al. valuate vicarious actions by incorporating within *Q*(S1) an action valuation for S1 which substitutes for *p*(S1). Actions and stimuli are, therefore, not dissociated as they are for the prospective route of the ATP network—the actions elicited by E1/E2 *do not* have “knowledge” of the stimulus, which permits the classification of *a number of* stimuli by affective value to then be associated with particular actions critical for TOC effects to manifest.

**Figure 10:**
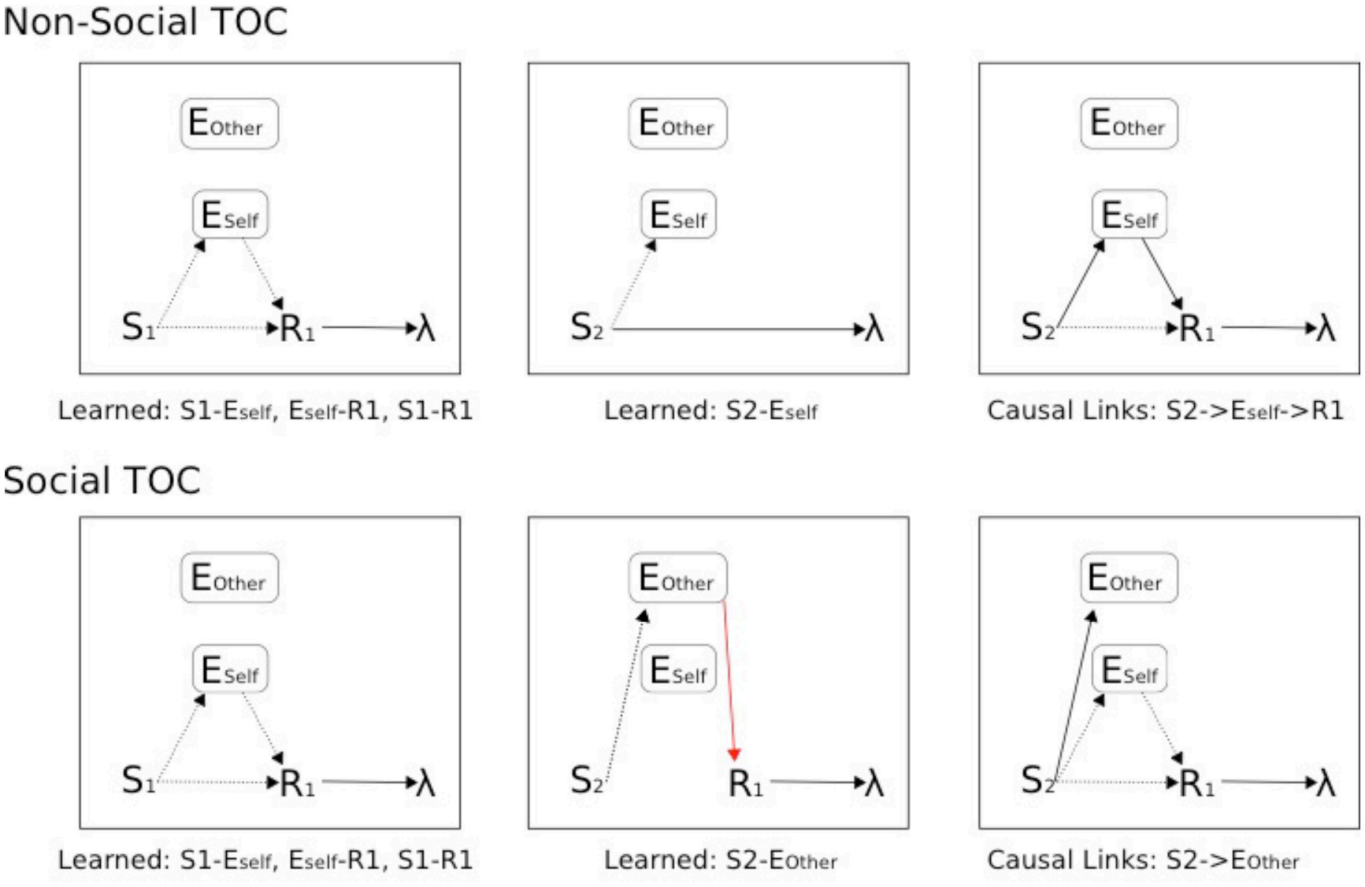
**Schematic of Associative Two-Process theoretic description of Pavlovian-Instrumental Transfer (TOC)**. **Top**: Non-Social TOC (standard ATP description of TOC). **Bottom**: Social TOC, ATP description according to our Suzuki et al. (2012) compatible social-valuation-specific ATP mechanism (Figure 9). Dashed lines represent learnable connections, Solid lines represent causal links, red solid lines represent links of the Other that are hidden to the Self. The three panels (left to right) concern instrumental, pavlovian, and instrumental transfer phase, respectively.

The ATP-based circuitry here (Figures 9, 10) focuses on what would be required for transfer of pavlovian knowledge from Other to Self, i.e., for our Social Aff-ATP hypothesis to hold. Importantly, from the perspective of a Social TOC, the network above-described (Figure 9) would *not* allow for transfer from Other to Self of the learned Stimulus-(Outcome) Expectancy maps in the instrumental transfer phase. This is because although it may be possible to learn the Other's (Social) value function (stimulus outcome valuations) in the pavlovian phase, the association between *Other's outcome expectation* and *Self response* cannot be made in the initial instrumental phase as sE1/sE2 outputs would have separate associations with actions / choices to E1/E2 outputs. This description is schematized in Figure 10. It is arguable as to whether the SVS-ATP mechanism depicted in Figure 9, would be more representative of the Suzuki et al. model if Social value magnitude and omission representations/nodes had direct inputs to the Non-Social equivalent nodes. A Social TOC would indeed, in this case, transpire. It would also make the Social value representation redundant when not tied to separate (simulated Other) actions. We have suggested that the SVS-ATP network would be useful when individuals wish to compare their valuations with those simulated for others and the actions they expect others to make in comparison to themselves. This might be viewed in terms of a competitive interaction scenario, but could also be useful in a Joint Action scenario where complementarity of other's valuations and actions to the self should often occur.

In Figure 10, the standard TOC (non-social/individualistic) is schematized along with the learned associations in each of the first two stages and the causal links that are exploited in the final (instrumental transfer) phase. This is a simplified set-up since a standard TOC would include multiple S-R mappings in each phase (allowing for differential outcomes classification of stimuli). The Social TOC, using the SVS-ATP mechanism (Figure 9) shows how such a transfer would not be possible. In the instrumental transfer phase, there is no learned (causal) link between the valuation of S2 for Other (*Eother*) and the response and so there is not a complete prospective route by which the correct response (R1) can be automatically cued (i.e., without further learning being necessary). Since the (Self) subject has not previously learned an S2-R1 association (via the retrospective route), there is no causal route to the correct response. Only if there is a further link between *Eother* and *Eself* value representations could a transfer be possible. The Social Aff-ATP mechanism (Figure 8), however, in utilizing the value function of Self for stimuli relevant to Other through vicarious stimulus processing, should re-produce the standard TOC found in individuals (Figure 10, top).

In summary, the Social Aff-ATP neural computational hypothesis would predict TOC effects that have been neural-computationally found using a model capturing data for an individual task. This mechanism conforms to the ECC perspective of Ruff and Fehr (2014). An alternative mechanism conforming to the SVS alternative perspective put forward by Ruff and Fehr (2014), and likened to the reinforcement learning model of Suzuki et al. (2012), should not produce a *social* TOC.

## Discussion

In this article, we have posited a neural computational hypothesis for a minimalist affective-learning mechanism for use in Joint Action. We have called this the Social Aff-ATP (neural computational) hypothesis, which provides a specific, testable implementation of the ECC hypothesis (cf. Ruff and Fehr, 2014). We discussed the ATP theory of differential outcomes learning. We then discussed our neural-computational modeling of this process and how a tweak of the model allowing for the incorporation of social stimuli inputs provides a social variant of the model. We also suggested an alternative mechanism that implements a SVS mechanism comparable to that of Suzuki et al.'s with separate social and non-social value functions. We have presented a schematic describing why this SVS-ATP implementation would not permit a social transfer of control (TOC) of the type that typically manifests in non-social contexts.

In the remainder of the Discussion Section, we will reiterate and elaborate on why we think our Social Aff-ATP mechanism constitutes a minimal mechanism that can have a useful function in Joint Action.

Vesper et al. (2010) has suggested that a feature of monitoring and detecting others' actions and action outcomes during Joint Action is to facilitate sensorimotor coordination during the Joint Action. Prediction can “smooth coordination” by enabling co-actors to accommodate each other in space and time or to compensate for deficiencies in performance of the other. Michael (2011) posited that emotions can provide such a role serving to facilitate alignment and monitoring and detecting of others (e.g., when the other expresses frustration). A perspective of Urcuioli (2005, 2013) is that outcome expectancies provide a means to effectively classify stimuli (see Figure 3). Action selection can then be simplified through exploiting affordances of the subset of those actions already associated with the outcome expectancy classes. This is a reason why participants under differential outcomes TOC training can immediately select the unique action that leads to the desired outcome even though the stimulus-action (response) contingency has previously not been experienced: *Subjects have already classified the stimuli according to a given outcome expectancy previously associated with an action*. This of course depends on a passive pavlovian phase. We conjecture from an evolutionary perspective it is natural that such observation might be exploited in a social context. In this case, agents observing the stimulus context of another (con-specific), irrespective of strong monitoring of actions, can learn from the stimulus-outcome contingencies and, via ECC circuitry, bring to bear such knowledge on their own instrumental capabilities. Such an ability, facilitates coordination as it subverts the need for a ponderous learning process during the Joint Action. Thereby, particularly when Joint Action is of a sensor-motorically complex nature or requires rapid and flexible interactions a Social Aff-ATP mechanism may reduce the monitoring of the other's (behavioral) activity. It may often suffice to be aware of the (stimulus) context and the (affective) outcomes of the Other.

The affective component of ATP, which concerns outcome expectancy classifications for differential dimensions of rewards (e.g., differing reward magnitudes, presentation/omission probabilities, qualitatively different rewards) or punishers, may be particularly pertinent to Joint Action. The affective properties of emotion contagion, and empathy identified by Michael (2011) are particularly relevant. In the case of the former, an actor may align his/her affective (outcome expectancy) state with the co-actor thereby cuing a subset of action possibilities similar to those cued in the observed actor. In this case, observation of (affective) outcomes may not be necessary to “smooth” coordination but rather observation of the expression of the other when it is isomorphic with the other's affective state. This expression can thereby be predictive of the outcome and facilitate (corrective) action in anticipation of (undesired) desired outcomes. We could envisage social stimuli—face and other bodily expressive computed inputs—as providing an input to the value function alternatives depicted in Figure 8 (social Aff-ATP) and Figure 9 (SVS-ATP) in this case.

The case of empathy relates to our Social Aff-ATP hypothesis where perception of stimuli, in the context of the presence of another, innervates circuits (e.g., mirror neuron circuits) that relate to one's own affective experience. Empathy and contagion may draw on related neural circuits (cf. De Vignemont and Singer, 2006), which recruit similar neural structures as those alluded to for our Social Aff-ATP (see Section Neural-Computational Basis for Affective Valuation in Individual Action)—and also in Knoblich and Jordan (2002). Bernhardt and Singer (2012) (see also Decety, 2011), for example, advocate a core (value-based) circuitry involving amygdala, vmPFC, dopaminergic pathways, as well as anterior cingulate cortex and insula.

Joint Action is, of course, a dynamic phenomenon, and it might be contended that it is not captured using a minimalist, turn-taking, procedure such as the differential outcomes TOC procedure. Such a controlled approach, however, allows for relative analytic ease in identifying mechanisms that may be used in the “wild.” A means of further bridging this turn-taking minimalist approach with a more dynamic method would be to include parallel learning and action selection (in line with the minimalist approaches of Sebanz et al., 2003, 2005; Atmaca et al., 2011; Loehr and Vesper, 2016). It is, in fact, possible to employ TOC procedures where the pavlovian and transfer phase are run concurrently (Overmier and Lawry, 1979). Turn-taking TOC procedures might also be used with fine resolution interleaving, e.g., switching from (social) pavlovian to transfer trials every other trial as compared to switching following a block of (social) pavlovian-only or transfer-only trials. Neural-dynamic computational models, of the type we have proposed in this article, may also be deployed in controlled but continuous Joint Action settings when used as controllers for artificial agents, e.g., virtual agents in virtual environments.

Notwithstanding the above arguments for appropriate testing of a social TOC, it is critical to appropriately evaluate whether subjects are, in fact, perceiving stimuli as social (e.g., vicarious) or not (ego-centric). Suzuki et al. (2012) employed sophisticated behavioral analytic means to suggest subjects did indeed act vicariously in choosing reward for others (given that they were themselves rewarded for correct Other choice predictions). Social valuation circuitry may even dynamically express itself as more or less ECC-based or SVS-based depending on the interactive nature of the task (note, Nicolle et al., 2012 found that different circuits might be deployed dynamically according to changing interactive scenarios). Neural representations for individual Self/Other and joint (action) effects may similarly entail dynamic expression according to changing perceptions of the social context in which the agents are acting.

## Author contributions

RL conceived of the model and contributed most of the written content. CV and JM provided background theories, discussion and feedback upon which the model and novel content was based. AA, GL, and PG contributed discussions, written feedback, and written content to the article.

### Conflict of interest statement

The authors declare that the research was conducted in the absence of any commercial or financial relationships that could be construed as a potential conflict of interest.

## Figures and Tables

**Figure A1:**
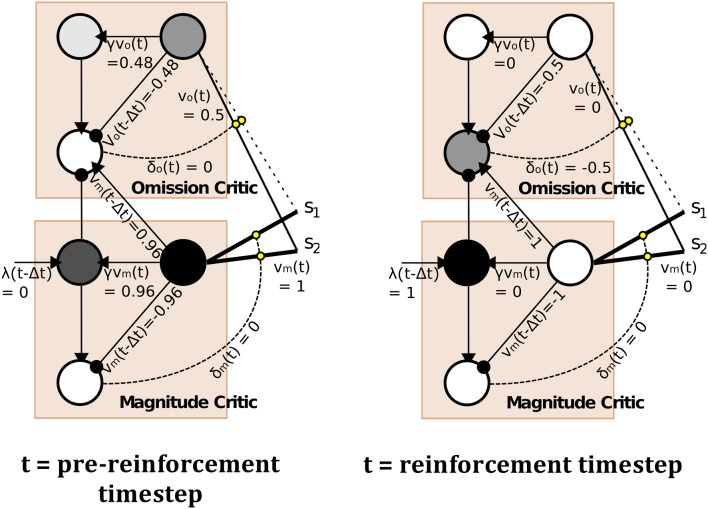
**Differential outcomes computations**. Computations of the Critic following learning of the value of a particular stimulus (0.5 omission probability of reinforcement of magnitude value 1.0). **Left**: At the time step prior to reinforcer presentation. **Right**: At the timestep of reinforcer presentation. Darker nodes represent greater activation; black, full activation of 1; white, zero activation. See Equations (A1, A2, A6, A7) for computation equations and text for explanation.

## Footnotes

^1^Shared goals here are defined as “complexes of states and relations” (p. 13).

^2^Such activity is not considered Joint Action since the participants' behavior is not required to be in any way adapted to each other in order to achieve the desired outcome.

^3^A distinction between affective and emotional states can be made on the basis that the latter may be considered a subset of the former which includes phenomena such as moods, drives, motivations.

^4^“Correct” means that the response, given a particular stimulus, is that which provides some kind of positive feedback either because it concerns adherence to an explicit task rule, or because it leads to a rewarding or interesting outcome.

^5^Alternatively, this behavior could be described as “best guess based on existing evidence.”

^6^The above only offers a role for our ATP neural-computational model in reward-based learning. In relation to punishment, the simplest assumption would be that a mirroring of the reward process occurs for punishment acquisition and omission/termination. Such mirroring systems have previously been modeled with respect to reward (acquisition) and punishment (acquisition), e.g., Daw et al. (2002), Alexander and Sporns (2002). Such a straightforward mirroring for punishment systems is, however, considered unlikely by many neuroscientists (e.g., Boureau and Dayan, 2010).

^7^This architecture extends that of Bicho et al. (2011) described above by introducing an additional “Emotional State Layer” of neural computational units that provide inputs into a module of units for intention perception of other.

^8^Correspondent activity, of course, needn't imply that such structures provide the root of such activity.

## Appendix

### Appendix A

Our model is given by Equations (A1–A7).

(A1) $$V_{e}\left(t\right)=\sum_{s\epsilon S}\;\left[\theta_{e_{s}}(t)\phi_{s}(t)\right]$$

where *V*_*e*_(*t*) is the learned value function; θ_*e*_*s*__(*t*) implements the value function update rule (Equation A2); *e* ϵ {*m, o*} is an index denoting Magnitude or Omission value functions, respectively; *t* is time in [1, *T*] where *T* is the total time over which a temporal representation of stimulus (*s*) memory is presented to the network; *s* is the number of different (social or non-social) stimuli in [1, *S*] where *S* = 2 for most cases of differential outcomes training.

(A2) $$\theta_{e}\left(t\right)=\theta_{e}\left(t-\Delta t\right)\;+\;\beta_{e}\sigma_{e}\phi_{s}\;(t-\Delta t)$$

where β_*e*_ is a learning rate in [0, 1]; Δ*t* is the time window; σ_*e*_ is the prediction error term; ϕ_*s*_ is calculated as ϕ_*s*_ (*t*) = ϕ_*s*_ (*t* − Δ*t*)γλ_*TD*_, where $\gamma =1-\frac{\Delta t}{\tau }$ and λ_*TD*_ = 1, implementing TD(1).

(A3) $$E_{2}\left(t\right)=\Lambda \;(V_{o}\;\left(t\right))$$

(A4) $$E_{1}\left(t\right)=\Lambda (V_{m}\;\left(t\right)-E_{2}\;(t))$$

(A5) $$\Lambda \left(x\right)=\frac{1}{1+exp\left[-\beta (x-\theta )\right]}$$

Equations (A3–A5) implement the output nodes computations of the network. *E*_2_(*t*) provides the output of *V*_*o*_(*t*) and *E*_1_(*t*) provides the output of *V*_*m*_(*t*). The sigmoid function in Equation (A5) transforms the outputs of the *V*_*m*_(*t*) and *V*_*o*_(*t*) value functions to effectively produce expectations (or classifications) of stimuli inputs according to reward acquisition, and reward omission expectation, respectively.

(A6) $$\sigma_{m}\left(t\right)=\lambda \left(t-\Delta t\right)+\frac{1}{\Delta t}\left[\left(1-\frac{\Delta t}{\tau }\right)V_{m}\left(t\right)-V_{m}(t-\Delta t)\right]$$

(A7) $$\sigma_{o}\left(t\right)=-\sigma_{m}\left(t\right)+\frac{1}{\Delta t}\left[\left(1-\frac{\Delta t}{\tau }\right)V_{o}\left(t\right)-V_{o}(t-\Delta t)\right]$$

In Equation (A6) Doya's (2000) continuous time TD learning prediction error (for the magnitude value function) is given. λ(*t* − Δ*t*) is the reinforcer input (stimulus) λ *ϵ* {0|1}, τ > Δ*t* is the decay constant. Equation (A7) in our model provides the omission value function prediction error σ_*o*_ (*t*), which subtracts the error of magnitude value function σ_*m*_ (*t*) and computes its own temporally discounted prediction error for expected omission. The same equations may be used both for our individual ATP model (Section Associative Two-Process Theory and Affect) and for our social ATP model (Section Social Valuation and ATP). We do not provide specific values for variables as the social ATP model currently provides our hypothesis to be evaluated.

### Appendix B

In Figure A1 is depicted the computational process of omission probability, using the prediction error rule of Equation (A7), that is the novel feature of our TD model. The network shows the activation levels at the time step preceding reinforcement presentation (Figure A1, left), and at the time step at reinforcement presentation (Figure A1, right). These computations show reinforcement predictions of an external stimulus, via prediction error updating, to provide a magnitude value of 1.0 and an omission probability value of 0.5. Figure A1 (left) shows, following learning, both the magnitude and omission values (*V*_*m*_(*t*) and *V*_*o*_(*t*), respectively) of the reinforcer when it is perfectly predicted. The prediction nodes of magnitude and omission critic are set to zero (white circles) as the inputs from the magnitude value node (black circle) is perfectly temporally predicted via the relayed discounted value node (lighter gray circle symbolizing a lower value) which receives input γ*V*_*m*_(*t*). When reinforcement is presented (λ(*t* − Δ*t*) = 1, in Figure A1 right) the input from the magnitude value node to the prediction error node at the previous timestep *V*_*m*_(*t* − Δ*t*) = −1) cancels the effect of the reinforcement input (λ(*t* − Δ*t*)). However, for the Omission Critic, there is a 0.5 prediction of omission and since reinforcement presentation (of magnitude 1) cancels the *V*_*m*_(*t* − Δ*t*) input, this produces a −0.5 prediction error and reduces the omission value weights according to its update rate (Equation A7). For this reason, prediction of omission probability will fluctuate around 0.5 following presentations of expected, or omitted, reinforcement as a function of the rate of learning—the smaller, the less fluctuation. Thus, when reinforcement is unexpectedly omitted (not shown), nothing reduces the previous timestep *V*_*m*_(*t* − Δ*t*) = 1 input so the Omission Critic prediction error node now generates an error of +0.5. The magnitude value, being learned, is not affected by the negative prediction error as there is no unlearning of the intrinsic magnitude of the reinforcer. Both Omission Critic and Magnitude Critic learn by their temporal difference learning rules producing asymptotically growing value functions from Stimulus onset time until reinforcer presentation time.

## References

[B1] Adolphs R. (2010). Conceptual challenges and directions for social neuroscience. Neuron 65, 752–767. 10.1016/j.neuron.2010.03.006 20346753 PMC2887730

[B2] Alexander W. Sporns O. (2002). An embodied model of learning, plasticity, and reward. Adapt. Behav. 10, 143–159. 10.1177/1059712302010003001

[B3] Atmaca S. Sebanz N. Knoblich G. (2011). The joint flanker effect: sharing tasks with real and imagined co-actors. Exp. Brain Res. 211, 371–385. 10.1007/s00221-011-2709-9 21573746 PMC3102196

[B4] Balkenius C. Morén J. (2001). Emotional learning: a computational model of the amygdala. Cybernet. Syst. 32, 611–636. 10.1080/01969720118947

[B5] Balkenius C. Morén J. Winberg S. (2009). Interactions between motivation, emotion and attention: from biology to robotics, in Proceedings of the Ninth International Conference on Epigenetic Robotics, Vol. 149 (Lund University Cognitive Studies).

[B6] Behrens T. E. Woolrich M. W. Walton M. E. Rushworth M. F. (2007). Learning the value of information in an uncertain world. Nat. Neurosci. 10, 1214–1221. 10.1038/nn1954 17676057

[B7] Bernhardt B. C. Singer T. (2012). The neural basis of empathy. Annu. Rev. Neurosci. 35, 1–23. 10.1146/annurev-neuro-062111-150536 22715878

[B8] Bicho E. Erlhagen W. Louro L. e Silva E. C. (2011). Neuro-cognitive mechanisms of decision making in joint action: a human–robot interaction study. Hum. Mov. Sci. 30, 846–868. 10.1016/j.humov.2010.08.012 21208673

[B9] Boureau Y.-L. Dayan P. (2010). Opponency revisited: competition and cooperation between dopamine and serotonin. Neuropsychopharmacology 1, 1–24. 10.1038/npp.2010.151 PMC3055522 20881948

[B10] Brass M. Heyes C. (2005). Imitation: is cognitive neuroscience solving the correspondence problem? Trends Cogn. Sci. 9, 489–495. 10.1016/j.tics.2005.08.007 16126449

[B11] Bratman M. E. (1992). Shared cooperative activity. Philos. Rev. 101, 327–341. 10.2307/218553720881948

[B12] Burke C. J. Tobler P. N. Baddeley M. Schultz W. (2010). Neural mechanisms of observational learning. Proc. Natl. Acad. Sci. U.S.A. 107, 14431–14436. 10.1073/pnas.1003111107 20660717 PMC2922583

[B13] Butterfill S. (2012). Joint action and development. Philos. Q. 62, 23–47. 10.1111/j.1467-9213.2011.00005.x

[B14] Cacioppo J. T. Berntson G. G. (1992). Social psychological contributions to the decade of the brain: doctrine of multilevel analysis. Am. Psychol. 47:1019. 10.1037/0003-066X.47.8.1019 1510329

[B15] Damasio A. R. (1994). Descartes' Error: Emotion, Reason, and the Human Brain. New York, NY: GP Putnam's Sons.

[B16] Damasio A. R. (1999). The Feeling of What Happens: Body, Emotion and the Making of Consciousness. London: Vintage.

[B17] Daw N. D. Kakade S. Dayan P. (2002). Opponent interactions between serotonin and dopamine. Neural Netw. 15, 603–616. 10.1016/S0893-6080(02)00052-7 12371515

[B18] Decety J. (2011). The neuroevolution of empathy. Ann. N.Y. Acad. Sci. 1231, 35–45. 10.1111/j.1749-6632.2011.06027.x 21651564

[B19] de Guzman M. Bird G. Banissy M. J. Catmur C. (2016). Self–other control processes in social cognition: from imitation to empathy. Philos. Trans. R. Soc. B Sci. 371:20150079. 10.1098/rstb.2015.0079 26644597 PMC4685524

[B20] Delamater A. R. (2007). The role of the orbitofrontal cortex in sensory-specific encoding of associations in pavlovian and instrumental conditioning. Ann. N.Y. Acad. Sci. 1121, 152–173. 10.1196/annals.1401.030 17872387

[B21] De Vignemont F. Singer T. (2006). The empathic brain: how, when and why? Trends Cogn. Sci. 10, 435–441. 10.1016/j.tics.2006.08.008 16949331

[B22] de Waal F. B. (2008). Putting the altruism back into altruism: the evolution of empathy. Annu. Rev. Psychol. 59, 279–300. 10.1146/annurev.psych.59.103006.093625 17550343

[B23] de Wit S. Dickinson A. (2009). Associative theories of goal-directed behaviour: a case for animal–human translational models. Psychol. Res. 73, 463–476. 10.1007/s00426-009-0230-6 19350272 PMC2694930

[B24] Doya K. (2000). Reinforcement learning in continuous time and space. Neural Comput. 12, 219–245. 10.1162/089976600300015961 10636940

[B25] Esteban L. Plaza V. López-Crespo G. Vivas A. B. Estévez A. F. (2014). Differential outcomes training improves face recognition memory in children and in adults with Down syndrome. Res. Dev. Disabil. 35, 1384–1392. 10.1016/j.ridd.2014.03.031 24713518

[B26] Estévez A. F. Fuentes L. J. Marí-Beffa P. González C. Alvarez D. (2001). The differential outcome effect as a useful tool to improve conditional discrimination learning in children. Learn. Motiv. 32, 48–64. 10.1006/lmot.2000.1060

[B27] Estévez A. F. Overmier J. B. Fuentes L. J. (2003). Differential outcomes effect in children: demonstration and mechanisms. Learn. Motiv. 34, 148–167. 10.1016/S0023-9690(02)00510-6

[B28] Friston K. Frith C. (2015). A duet for one. Conscious. Cogn. 36, 390–405. 10.1016/j.concog.2014.12.003 25563935 PMC4553904

[B29] Frith C. D. Wentzer T. S. (2013). Neural hermeneutics, in Encyclopedia of Philosophy and the Social Sciences, ed Kaldis B. (Thousand Oaks, CA: SAGE Publications, Inc.), 657–659.

[B30] Gilbert M. (1990). Walking together: a paradigmatic social phenomenon. Midwest Stud. Philos. 15, 1–14. 10.1111/j.1475-4975.1990.tb00202.x

[B31] Heyes C. Bird G. (2008). Mirroring, association, and the correspondence problem. Sensorimot. Found. High. Cogn. 22, 461 10.1093/acprof:oso/9780199231447.001.0001

[B32] Hikosaka K. Watanabe M. (2000). Delay activity of orbital and lateral prefrontal neurons of the monkey varying with different rewards. Cereb. Cortex 10, 263–271. 10.1093/cercor/10.3.263 10731221

[B33] Hikosaka K. Watanabe M. (2004). Long- and short-range reward expectancy in the primate orbitofrontal cortex. Eur. J. Neurosci. 9, 1046–1054. 10.1111/j.0953-816X.2004.03120.x 15009152

[B34] Holden J. M. Overmier J. B. (2014). Performance under differential outcomes: contributions of reward-specific expectancies. Learn. Motiv. 45, 1–14. 10.1016/j.lmot.2013.09.001

[B35] Holden J. M. Overmier J. B. (2015). Choice behavior under differential outcomes: sample stimulus control versus expectancy control. Learn. Motiv. 51, 50–61. 10.1016/j.lmot.2015.04.002

[B36] Houk J. C. Adams J. L. Barto A. G. (1995). A model of how the basal ganglia generate and use neural signals that predict reinforcement, in Models of Information Processing in the Basal Ganglia, eds Houk J. C. Davis J. L. Beiser D. G. (Cambridge, MA: MIT Press), 249–270.

[B37] Knoblich G. Jordan S. (2002). The mirror system and joint action, in Mirror Neurons and the Evolution of Brain and Language, eds Stamenov M. I. Gallese V. (Amsterdam: John Benjamins), 115–124.

[B38] Kruse J. M. Overmier J. B. (1982). Anticipation of reward omission as a cue for choice behavior. Learn. Motiv. 13, 505–525. 10.1016/0023-9690(82)90007-8

[B39] Loehr J. D. Vesper C. (2016). The sound of you and me: novices represent shared goals in joint action. Q. J. Exp. Psychol. 69, 535–547. 10.1080/17470218.2015.1061029 26073040

[B40] Lowe R. Sandamirskaya Y. Billing E. (2014). A neural dynamic model of associative two-process theory: the differential outcomes effect and infant development, in Development and Learning and Epigenetic Robotics (ICDLEpirob) (Genoa), 440–447.

[B41] Maki P. Overmier J. B. Delos S. Gumann A. J. (1995). Expectancies as factors influencing conditional discrimination performance of children. Psychol. Rec. 45, 45–71.

[B42] McDannald M. A. Saddoris M. P. Gallagher M. Holland P. C. (2005). Lesions of orbitofrontal cortex impair rats differential outcome expectancy learning but not conditioned stimulus-potentiated feeding. J. Neurosci. 25, 4626–4632. 10.1523/JNEUROSCI.5301-04.2005 15872110 PMC1201522

[B43] Miceli M. Castelfranchi C. (2014). Expectancy and Emotion. Oxford: Oxford University Press.

[B44] Michael J. (2011). Shared emotions and joint action. Rev. Philos. Psychol. 2, 355–373. 10.1007/s13164-011-0055-2

[B45] Miller S. (1992). Joint action. Philos. Pap. 21, 275–297. 10.1080/055686492095063866490077

[B46] Milward S. J. Kita S. Apperly I. A. (2014). The development of co-representation effects in a joint task: do children represent a co-actor? Cognition 132, 269–279. 10.1016/j.cognition.2014.04.008 24853630

[B47] Milward S. J. Sebanz N. (2016). Mechanisms and development of self–other distinction in dyads and groups. Philos. Trans. R. Soc. B Sci. 371:20150076. 10.1098/rstb.2015.0076 26644595 PMC4685522

[B48] Morén J. (2002). Emotion and Learning: A Computational Model of the Amygdala. Ph.D. thesis, Lund University.

[B49] Nicolle A. Klein-Flügge M. Hunt L. T. Vlaev I. Dolan R. Behrens T. (2012). An agent independent axis for executed and modeled choice in medial prefrontal cortex. Neuron 75, 114–1121. 10.1016/j.neuron.2012.07.023 22998878 PMC3458212

[B50] Overmier J. B. Lawry J. A. (1979). Pavlovian conditioning and the mediation of behavior, in The Psychology of Learning and Motivation, Vol. 13, ed Bower G. H. (New York, NY: Academic Press), 1–55.

[B51] Passingham R. E. Wise S. P. (2012). The Neurobiology of the Prefrontal Cortex: Anatomy, Evolution, and the Origin of Insight (No. 50). Oxford: Oxford University Press.

[B52] Peterson G. B. Trapold M. A. (1982). Expectancy mediation of concurrent conditional discriminations. Am. J. Psychol. 95, 571–580. 10.2307/1422188 7168456

[B53] Redgrave P. Prescott T. J. Gurney K. (1999). Is the short-latency dopamine response too short to signal reward? Trends Neurosci. 22, 146–151. 10.1016/S0166-2236(98)01373-3 10203849

[B54] Richardson D. C. Street C. N. Tan J. Y. Kirkham N. Z. Hoover M. A. Ghane Cavanaugh A. (2012). Joint perception: gaze and social context. Front. Hum. Neurosci. 6:194. 10.3389/fnhum.2012.00194 22783179 PMC3388371

[B55] Rolls E. T. (1999). The Brain and Emotion. Oxford: Oxford University Press.

[B56] Rolls E. T. (2013). What are emotional states, and why do we have them? Emot. Rev. 5, 241–247. 10.1177/1754073913477514

[B57] Ruff C. C. Fehr E. (2014). The neurobiology of rewards and values in social decision making. Nat. Rev. Neurosci. 15, 549–562. 10.1038/nrn3776 24986556

[B58] Sacheli L. M. Aglioti S. M. Candidi M. (2015). Social cues to joint actions: the role of shared goals. Front. Psychol. 6:1034. 10.3389/fpsyg.2015.01034 26283986 PMC4519671

[B59] Schoenbaum G. Chiba A. A. Gallagher M. (1998). Orbitofrontal cortex and basolateral amygdala encode expected outcomes during learning. Nat. Neurosci. 1, 155–159. 10.1038/407 10195132

[B60] Schoenbaum G. Saddoris M. P. Stalnaker T. A. (2007). Reconciling the roles of orbitofrontal cortex in reversal learning and the encoding of outcome expectancies. Ann. N.Y. Acad. Sci. 1121, 320–335. 10.1196/annals.1401.001 17698988 PMC2430624

[B61] Schoenbaum G. Setlow B. Saddoris M. P. Gallagher M. (2003). Encoding predicted outcome and acquired value in orbitofrontal cortex during cue sampling depends upon input from basolateral amygdala. Neuron 39, 855–867. 10.1016/S0896-6273(03)00474-4 12948451

[B62] Schultz W. (1998). Predictive reward signal of dopamine neurons. J. Neurophysiol. 80, 1–27. 9658025 10.1152/jn.1998.80.1.1

[B63] Schultz W. (2007). Multiple Dopamine functions at different time courses. Annu. Rev. Neurosci. 30, 259–288. 10.1146/annurev.neuro.28.061604.135722 17600522

[B64] Searle J. R. (1990). Collective intentions and actions. Intent. Commun. 401, 401–402.

[B65] Sebanz N. Bekkering H. Knoblich G. (2006). Joint action: bodies and minds moving together. Trends Cogn. Sci. 10, 70–76. 10.1016/j.tics.2005.12.009 16406326

[B66] Sebanz N. Knoblich G. (2009). Prediction in joint action: what, when, and where. Top. Cogn. Sci. 1, 353–367. 10.1111/j.1756-8765.2009.01024.x 25164938

[B67] Sebanz N. Knoblich G. Prinz W. (2003). Representing others actions: just like one's own? Cognition 88, B11–B21. 10.1016/S0010-0277(03)00043-X 12804818

[B68] Sebanz N. Knoblich G. Prinz W. (2005). How two share a task: corepresenting stimulus-response mappings. J. Exp. Psychol. Hum. Percept. Perform. 31, 1234–1246. 10.1037/0096-1523.31.6.1234 16366786

[B69] Silva R. Louro L. Tiago M. Erlhagen W. Bicho E. (in press). Combining intention and emotional state inference in a dynamic neural field architecture for human-robot joint action, in Special Issue on Grounding Emotions in Robots: Embodiment, Adaptation, Social Interaction, Adaptive Behavior, eds Lowe R. Barakova E. Billing E. Broekens J. (Sage).

[B70] Steinbeis N. (2016). The role of self–other distinction in understanding others' mental and emotional states: neurocognitive mechanisms in children and adults. Philos. Trans. R. Soc. B Sci. 371:20150074. 10.1098/rstb.2015.0074 26644593 PMC4685520

[B71] Sutton R. S. Barto A. G. (1998). Reinforcement Learning: An Introduction. Cambridge, MA: The MIT Press.

[B72] Suzuki S. Harasawa N. Ueno K. Gardner J. L. Ichinohe N. Haruno M.. (2012). Learning to simulate others' decisions. Neuron 74, 1125–1137. 10.1016/j.neuron.2012.04.030 22726841

[B73] Tomasello M. (2010). Origins of Human Communication. Cambridge, MA: MIT Press.

[B74] Tomasello M. Carpenter M. Call J. Behne T. Moll H. (2005). Understanding and sharing intentions: the origins of cultural cognition. Behav. Brain Sci. 28, 675–691. 10.1017/S0140525X05000129 16262930

[B75] Trapold M. A. (1970). Are expectancies based upon different positive rein- forcing events discriminably different? Learn. Motiv. 1, 129–140. 10.1016/0023-9690(70)90079-2

[B76] Tuomela R. (1993). What is cooperation? Erkenntnis 38, 87–101. 10.1007/BF0112902326786690

[B77] Urcuioli P. J. (1990). Some relationships between outcome expectancies and sample stimuli in pigeons delayed matching. Anim. Learn. Behav. 18, 302–314. 10.3758/BF03205290

[B78] Urcuioli P. J. (2005). Behavioral and associative effects of differential outcomes in discrimination learning. Anim. Learn. Behav. 33, 1–21. 10.3758/BF03196047 15971490

[B79] Urcuioli P. J. (2013). Stimulus control and stimulus class formation, in APA Handbook of Behavior Analysis, Vol. 1, eds Madden G. J. Dube W. V. Hackenberg T. D. Hanley G. P. Lattal K. A. (Washington, DC: American Psychological Association), 361–386.

[B80] Vesper C. Butterfill S. Knoblich G. Sebanz N. (2010). A minimal architecture for joint action. Neural Netw. 23, 998–1003. 10.1016/j.neunet.2010.06.002 20598504

[B81] Watanabe M. Hikosaka K. Sakagami M. Shirakawa S. (2007). Reward expectancy-related prefrontal neuronal activities: are they neural substrates of “affective” working memory? Cortex 43, 53–64. 10.1016/S0010-9452(08)70445-3 17334207

